# An Equine Model for Vaccination against a Hepacivirus: Insights into Host Responses to E2 Recombinant Protein Vaccination and Subsequent Equine Hepacivirus Inoculation

**DOI:** 10.3390/v14071401

**Published:** 2022-06-27

**Authors:** Marcha Badenhorst, Armin Saalmüller, Janet M. Daly, Reinhard Ertl, Maria Stadler, Christina Puff, Madeleine de le Roi, Wolfgang Baumgärtner, Michael Engelmann, Sabine Brandner, Hannah K. Junge, Barbara Pratscher, Asisa Volz, Bertrand Saunier, Thomas Krey, Johannes Wittmann, Steffen Heelemann, Julien Delarocque, Bettina Wagner, Daniel Todt, Eike Steinmann, Jessika-M. V. Cavalleri

**Affiliations:** 1 Clinical Unit of Equine Internal Medicine, Department for Companion Animals and Horses, University of Veterinary Medicine Vienna (Vetmeduni), Veterinärplatz 1, 1210 Vienna, Austria; marcha.badenhorst@vetmeduni.ac.at (M.B.); hjunge@vetclinics.uzh.ch (H.K.J.); barbara.pratscher@vetmeduni.ac.at (B.P.); 2 Department of Pathobiology, Institute of Immunology, University of Veterinary Medicine Vienna (Vetmeduni), Veterinärplatz 1, 1210 Vienna, Austria; armin.saalmueller@vetmeduni.ac.at (A.S.); maria.stadler@vetmeduni.ac.at (M.S.); 3 School of Veterinary Medicine and Science, University of Nottingham, Sutton Bonington, Leicestershire LE12 5RD, UK; janet.daly@nottingham.ac.uk; 4 VetCore Facility for Research, University of Veterinary Medicine Vienna (Vetmeduni), Veterinärplatz 1, 1210 Vienna, Austria; reinhard.ertl@vetmeduni.ac.at; 5 Institute of Pathology, University of Veterinary Medicine Hanover, 30559 Hanover, Germany; christina.puff@tiho-hannover.de (C.P.); madeleine.de.le.roi@tiho-hannover.de (M.d.l.R.); wolfgang.baumgaertner@tiho-hannover.de (W.B.); 6 Department of Molecular and Medical Virology, Faculty of Medicine, Ruhr University Bochum, 44801 Bochum, Germany; michael.engelmann@ruhr-uni-bochum.de (M.E.); daniel.todt@ruhr-uni-bochum.de (D.T.); 7 VetFarm, University of Veterinary Medicine Vienna (Vetmeduni), Kremesberg 13, 2563 Pottenstein, Austria; sabine.brandner@vetmeduni.ac.at; 8 Clinic for Equine Internal Medicine, Vetsuisse Faculty, University of Zurich, Winterthurerstrasse 260, 8057 Zurich, Switzerland; 9 Clinical Unit of Small Animal Internal Medicine, Department for Companion Animals and Horses, University of Veterinary Medicine Vienna (Vetmeduni), Veterinärplatz 1, 1210 Vienna, Austria; 10 Institute of Virology, University of Veterinary Medicine Hanover Foundation, 30559 Hanover, Germany; asisa.volz@tiho-hannover.de; 11 Structural Virology Unit, Institut Pasteur, Paris Cité University, CNRS UMR 3569, 75015 Paris, France; bertrand.saunier@pasteur.fr; 12 Center of Structural and Cell Biology in Medicine, Institute of Biochemistry, University of Lübeck, 23562 Lübeck, Germany; krey@biochem.uni-luebeck.de; 13 Center for Structural Systems Biology (CSSB), 22607 Hamburg, Germany; 14 German Center for Infection Research (DZIF), Partner Site Hamburg-Lübeck-Borstel-Riems, 23562 Lübeck, Germany; 15 Cluster of Excellence RESIST (EXC 2155), Hanover Medical School, 30625 Hanover, Germany; 16 Institute of Virology, Hanover Medical School, 30625 Hanover, Germany; 17 Lifespin GmbH, 93053 Regensburg, Germany; johannes.wittmann@lifespin.de (J.W.); steffen.heelemann@lifespin.de (S.H.); 18 Clinic for Horses, University of Veterinary Medicine Hanover, 30559 Hanover, Germany; julien.delarocque@tiho-hannover.de; 19 Department of Population Medicine and Diagnostic Sciences, College of Veterinary Medicine, Cornell University, Ithaca, NY 14853, USA; bw73@cornell.edu; 20 European Virus Bioinformatics Centre (EVBC), 07743 Jena, Germany

**Keywords:** horse, liver, RNA sequencing, immunoglobulins, hepacivirus A, vaccine, viral challenge, metabolomics

## Abstract

Equine hepacivirus (EqHV) is the closest known genetic homologue of hepatitis C virus. An effective prophylactic vaccine is currently not available for either of these hepaciviruses. The equine as potential surrogate model for hepacivirus vaccine studies was investigated, while equine host responses following vaccination with EqHV E2 recombinant protein and subsequent EqHV inoculation were elucidated. Four ponies received prime and booster vaccinations (recombinant protein, adjuvant) four weeks apart (day −55 and −27). Two control ponies received adjuvant only. Ponies were inoculated with EqHV RNA-positive plasma on day 0. Blood samples and liver biopsies were collected over 26 weeks (day −70 to +112). Serum analyses included detection of EqHV RNA, isotypes of E2-specific immunoglobulin G (IgG), nonstructural protein 3-specific IgG, haematology, serum biochemistry, and metabolomics. Liver tissue analyses included EqHV RNA detection, RNA sequencing, histopathology, immunohistochemistry, and fluorescent in situ hybridization. Al-though vaccination did not result in complete protective immunity against experimental EqHV inoculation, the majority of vaccinated ponies cleared the serum EqHV RNA earlier than the control ponies. The majority of vaccinated ponies appeared to recover from the EqHV-associated liver insult earlier than the control ponies. The equine model shows promise as a surrogate model for future hepacivirus vaccine research.

## 1. Introduction

Equine hepacivirus (EqHV) is one of 14 species belonging to the genus *Hepacivirus,* which also includes hepatitis C virus (HCV), in the family *Flaviviridae*—a genetically diverse group of human and animal pathogens [1,2]. EqHV is also referred to as canine hepacivirus, nonprimate hepacivirus and, most recently, *Hepacivirus* A [1]. The single stranded, ~9.2 kilobase (kb), positive-sense ribonucleic acid (RNA) genome of EqHV is the closest known genetic homologue of HCV [3,4]. EqHV and HCV do not only share common features of genomic structure but also biological properties [5]. Similar to HCV, experimental, blood-borne transmission of EqHV has been demonstrated, both chronic and acute courses of infection have been identified, and hepatotropism has been established with EqHV replication occurring exclusively in the liver [4,6,7,8]. 

An effective prophylactic vaccine is currently not available for either EqHV or HCV. Challenges that have hindered the development of an HCV vaccine include the sequence divergence of the virus, difficulties associated with HCV culture systems, limited models for testing vaccines, and an incomplete understanding of protective immune responses [9]. HCV host species tropism is restricted to humans and chimpanzees. A robust, immunocompetent animal model is lacking, hampering mechanistic analysis of virus pathogenesis, immune control, and prophylactic vaccine development. EqHV infection in horses provides an opportunity to study the host–pathogen interactions of a closely related hepacivirus in the mammalian host and the induction of a protective immune response [6]. The equine immune response has previously been suggested as a model for investigation of other infectious and allergic conditions in humans [10,11]. A distinct distribution of immunoglobulin (Ig) classes is observed in horses [10]. The 11 Ig isotypes include IgM, IgD, IgA, IgE, and seven IgG subclasses, named IgG1–IgG7 [12,13,14]. Separate genes encoding the constant heavy chain regions distinguish the different isotypes [15]. Comparable to humans, specific IgG subclass expression is associated with certain infections [10]. IgG4, IgG7 and IgG1 are primarily produced in response to intracellular pathogens, while IgG3 and IgG5 are primarily produced in response to extracellular pathogens [14].

Selecting antigens to maximize the induction of successful T-cell and immunoglobulin responses remains an area of active research. E2 is a membrane-anchored protein that interacts with multiple cell surface receptors, which mediate viral entry [9]. Despite the E2 envelope protein of HCV being among the most variable portions of its genome, it has remarkable sequence similarity to that of EqHV [3]. E2 is a target of the neutralizing immunoglobulin response against HCV [9]. Clearance of acute HCV infection and prevention of a chronic infection outcome have been associated with the timeous appearance of specific anti-E2 IgG isotypes [16]. Vaccination of rodents with recombinant HCV E2 alone induced polyclonal immunoglobulin responses with cross-reactive neutralizing activity [17]. Recombinant subunit vaccines are often less immunogenic than live-attenuated or inactivated vaccines and booster immunizations or the addition of adjuvants are strategies used to enhance the efficacy [18].

Thorough understanding of immune response mechanisms against these viruses may aid the future development of effective vaccines and optimal treatment strategies. Furthermore, investigations could lead to the development of new surrogate experimental models for HCV, exemplifying how innovative research at the interface of veterinary and medical science can benefit animals and humans alike. The aims of this investigation were to elucidate equine host responses following prime-boost vaccination with an EqHV E2 recombinant protein and subsequent EqHV inoculation, and to gain insight into an equine model for vaccination against a hepacivirus.

## 2. Materials and Methods

### 2.1. Experimental Animals

Six healthy, adult Shetland ponies (3 geldings, 3 mares), aged between 8 and 12 years, belonging to the University of Veterinary Medicine Vienna, were used in this study. Health status of the ponies was determined prior to commencement of the study, based on complete physical examination, haematology, and serum biochemistry. To ensure the negative EqHV-status of all ponies prior to commencement of the study, serum was analysed by luciferase immunoprecipitation system (LIPS) for the absence of anti-EqHV nonstructural protein 3 (NS3)-specific IgG, and by quantitative polymerase chain reaction (qPCR) for the absence of EqHV RNA. Additionally, serum samples from all ponies were confirmed to be PCR-negative for equine parvovirus hepatitis (EqPV-H) deoxyribonucleic acid (DNA).

### 2.2. EqHV E2 Recombinant Protein Production

A DNA sequence encoding *Drosophila* endoplasmic reticulum chaperone BiP leader peptide followed by EqHV E2 envelope protein ectodomain (sE2), enterokinase cleavage site and double Strep-tag II fusion protein (see amino acid sequence below) was inserted into a pECIA-14-like plasmid vector, the eukaryotic cell expression of which is driven by a metallothionein promoter. After co-transfection of *Drosophila melanogaster* S2 cells with this construct and an additional plasmid (molar ratio = 1/20) expressing puromycin resistance gene, the cells were grown in the presence of 5−10 µg/mL puromycin [19]. Stable transfectants were seeded at a density of 10^7^ cells/mL of Insect-X-press defined culture medium (Lonza Bioscience, Levallois-Perret, France); 1 L of the cell suspension was incubated at 28 °C in a 2-L spinner with magnetic stirrer and air bubbling systems (Corning, Corning, NY, USA) in the presence of 5 µM cadmium chloride (Merck-Sigma, Burlington, MA, USA). After 10 days, the cells were collected by centrifugation and culture supernatant was ultrafiltered through 10-kDa MWCO PES cassettes (Vivaflow 200, Sartorius); 1/10 volume of 1 M Tris-HCl, pH 8.0, was added to the concentrated supernatant fraction (flow-through is kept until purification is complete), as well as Avidin (Pierce, ThermoFisher Scientific, Waltham, MA, USA) at a final concentration of 15 µg/mL; to remove protein aggregates, the supplemented supernatant was subjected to centrifugation at higher than 20,000× *g* and 4 °C for 30 min. Resulting supernatant was then pump-loaded onto a 5-mL Strep-Tactin^®^ Superflow^®^ cartridge (IBA Lifesciences, Göttingen, Germany) according to manufacturer’s recommendations. After extensive affinity chromatography resin wash, purified EqHV sE2 protein was eluted with a solution containing 2.5 mM desthiobiotin (IBA Lifesciences). Recovered protein was further analyzed by size exclusion chromatography (Cytiva HiLoad™ Superdex 200, 16/600) driven by automated FPLC system (AKTApurifier^®^, GE Healthcare, Chicago, IL, USA). SEC fractions were analyzed by sodium dodecyl sulfate-polyacrylamide gel electrophoresis (SDS-PAGE) followed by Coomassie staining. sE2-positive fractions were concentrated with 10-kDa MWCO PES filters (Vivaspin 20, Sartorius, Göttingen, Germany) and finally sterile-filtered through 0.2-µM PES membranes (Millex^®^, Millipore, Burlington, MA, USA).

Amino acid sequence of sE2 fusion protein produced in this study **[leader peptide sequence is indicated in bold typeface]:**

**[MKLCILLAVVAFVGLSLG]**RSEASVVRAGGHIVSNDCNSSQILWAASDWAIHEVGCVPCVESTCWVPLTSSISVKNESVIVRGLGSHIDVLAAMASVCSTLGIGEACGAATLGYITFLSRFFMSLNLTNDCECFLYPGAISTFEFTMRALQSMMPNLSGFVSMFSGVPNTLFTIFTNGHWGVILALCLYGTTNNYFKLCLLLLAYSGLVSCESDYLNVSLSCNFTVKQMWGWTFFPKWAILNDQRLNCTEGSPYNPKCKGPMDFNITADPVIGYSGTRSHPPCPYHVSRPCSILDASRVCGKPTCFGPAPIEVGVTDQDGNLVSWNDSGKFFFDLRSPHRPPRGRWYGCVWLNSTGWVKQCGAPPCNMALMSGKGKTFVCPSDCFRQNPKATYQLCGQGPWISYNCLIDYTDRYLHFPCTENFTVYPVRMVLGDGARDVRVACKGPFEDDDDKAGWSHPQFEKGGGSGGGSGGGSWSHPQFEK

### 2.3. Vaccination and EqHV Inoculation

Random allocation was used to identify the “vaccine ponies” (*n* = 4; Vaccine Pony 1, 2, 3, and 4) and the “control ponies” (*n* = 2; Control Pony 1 and 2). Prime and booster vaccinations were administered four weeks apart, on day –55 and day –27. Vaccine ponies each received 100 µg EqHV E2 recombinant protein (Institut Pasteur, Paris, France) and 50 µg of the toll-like receptor 1 and 2 (TLR1/TLR2) ligand, Pam3Cys-GDPKHPKSF (*XS15*—EMC Microcollections GmbH, Tübingen, Germany), as adjuvant, diluted in phosphate-buffered saline (PBS) to a volume of 2 mL. Half of the vaccine volume was administered intramuscularly, and the other half subcutaneously. The same procedure was followed for the control ponies, although the control ponies received adjuvant only, without recombinant protein. On day 0, 27 days after the booster vaccination, all six ponies were inoculated intravenously with 10 mL of plasma from an EqHV PCR-positive, EqPV-H PCR-negative donor horse. The E2 recombinant protein used for vaccination (accession number JQ434001) and the E2 protein of the EqHV variant in the donor plasma (accession number KP739812) share amino acid sequence homology of 93.1%. 

### 2.4. Sample Collection

Over a period of 26 weeks, from day −70 until day +112, serum and whole blood samples were collected once per week by jugular venipuncture from all six ponies (except on day −63). Ultrasound-guided, percutaneous liver biopsies were collected from all six ponies at three time points: day −63 (reference samples), day +13 (early EqHV infection), and day +97 (late EqHV infection). 

### 2.5. Laboratory Analysis

#### 2.5.1. Detection of EqHV RNA in Serum

Serum samples were frozen at −80 °C after collection, prior to further processing at the Department of Molecular and Medical Virology, Ruhr University Bochum, Germany. Automated viral RNA extraction from 140 µL serum was done by using a QIAamp 12 Viral RNA Mini QIAcube Kit (Qiagen, Hilden, Germany) according to the manufacturer’s instructions. Extracted RNA (5 µL/reaction) was subjected to one-step SYBR Green-based qPCR using GoTaq^®^ qPCR Master Mix (Promega, Walldorf, Germany), in combination with the previously described primers targeting the 5ʹ untranslated region (5ʹUTR) [20]. A standard curve for the quantification of RNA copies was assessed by serial dilution of EqHV 5ʹUTR RNA based on the NPHV-NZP-1 (accession number JQ434001.1) sequence. Measurement of fluorescence was conducted with a LightCycler 480 Instrument II (Roche, Mannheim, Germany). The applied standard curve reached linearity at 1000 copies/reaction, resulting in a lower limit of quantitation (LLOQ) of 7.1 × 10^4^ viral copies/mL serum.

#### 2.5.2. Detection and Isotyping of Anti-EqHV E2-specific IgG (E2 IgG) in Serum 

Serum samples were frozen at −80 °C after collection, prior to further processing at the College of Veterinary Medicine, Cornell University, USA, for detection and isotyping of E2 IgG in a fluorescent-bead-based assay. The coupling of the recombinant EqHV E2 protein to fluorescent bead 33 (Luminex Corp., Austin, TX, USA) was performed as previously described in detail for other antigens [21]. A total of 40 μg of recombinant E2 antigen was used per 5 × 10^6^ beads. For the assay run, beads coupled with EqHV E2 were sonicated, mixed, and diluted in blocking buffer (PBS with 1% (*w*/*v*) bovine serum albumin (BSA) and 0.05% (*w*/*v*) sodium azide) to a final concentration of 1 × 10^5^ beads/mL each. All equine serum samples were tested undiluted. Millipore Multiscreen HTS plates (Millipore, Danvers, MA) were soaked with PBS-T (PBS with 0.1% (*w*/*v*) BSA, 0.02% (*v*/*v*) Tween 20 and 0.05% (*w*/*v*) sodium azide) using a ELx50 plate washer (Biotek Instruments Inc., Winooski, VT, USA) for 2 min. The solution was aspirated from the plates and 50 µL of each serum sample was applied to the plates. Subsequently, 50 µL of bead solution containing 5 × 10^3^ beads per well was added to the plate and incubated for 30 min on a shaker at room temperature. The plate was washed with PBS-T and 50 µL of biotinylated monoclonal anti-horse IgG1 (clone CVS45) added [22]. Additional plates with identical sample set-up were detected with biotinylated monoclonal antibodies against equine IgG4/7 (clone CVS39) [22], IgG3/5 (clone 586) [14], or IgG6 (clone 267) [14]. All detection antibodies were diluted 1:100 in blocking buffer and incubated for 30 min as above. After washing, 50 µL of streptavidin-phycoerythrin (Invitrogen, Carlsbad, CA, USA) diluted 1:100 in blocking buffer was added. Plates were incubated for 30 min as above and washed. The beads were resuspended in 100 µL of blocking buffer and each plate was placed on the shaker for 15 min to resuspend the beads. The assay was analysed in a Luminex 200 instrument (Luminex Corp., Austin, TX, USA) using BioPlex Manager 6.1 software (BioRad, Hercules, CA, USA). The data were reported as median fluorescent intensities (MFI).

#### 2.5.3. Detection of Anti-EqHV NS3-Specific IgG in Serum

Serum samples were frozen at −80 °C after collection, prior to further processing at the Department of Molecular and Medical Virology, Ruhr University Bochum, Germany, for LIPS analysis. Serum samples were analysed in duplicate for the presence of anti-EqHV NS3-specific IgG, using the LIPS assay as described previously [7,23]. Relative light units (RLU) were measured with a plate luminometer (LB 960 XS3; Berthold, Bad Wildbad, Germany). The threshold value, above which samples were regarded as NS3 IgG-positive, was calculated for each plate by using the mean value plus three standard deviations (SDs) of an EqHV-negative horse serum sample. 

#### 2.5.4. Monitoring of Haematology, Serum Biochemistry, and Clinical Parameters

Fresh whole blood and serum samples were analysed on the day of collection for haematology and serum biochemistry parameters at the Clinical Pathology Platform of the University of Veterinary Medicine Vienna. The laboratory’s reference ranges were used for interpretation of these data. Baseline values were determined on day –1, prior to viral inoculation, for all measured parameters except aspartate aminotransferase (AST). AST reference values were only available on day −70.

Rectal temperatures of the ponies were recorded every 24–48 h throughout the study period. Additionally, heart rate, respiratory rate, and rectal temperature were recorded immediately before and regularly after experimental interventions (liver biopsies, vaccination, and viral inoculation). 

Based on blood parameters (albumin, AST, GLDH, GGT, bilirubin, bile acids, triglycerides, serum iron, serum amyloid A, haematocrit, and leukocytes) and clinical parameters (heart rate, respiratory rate, and rectal temperature), an overall clinical score was calculated for each pony. Parameters that were evaluated, as well as the scores awarded, are listed in Supplementary Table S1. Clinical parameters recorded during the days subsequent to liver biopsies were excluded from the calculations, due to the potential confounding effect of the biopsy procedure on the clinical parameters. 

#### 2.5.5. ^1^H Nuclear Magnetic Resonance (NMR) Spectroscopy of Serum Samples

Serum samples were frozen at −80 °C after collection, prior to further processing at Lifespin GmbH, Regensburg, for metabolomic analysis. Metabolomic analysis was performed using NMR spectroscopy. Serum samples were thawed at room temperature and inverted five-fold; 350 µL serum and 350 µL aqueous buffer (H_2_O p.A., 0.1 g/L NaN_3_, 0.067 mol/L Na_2_HPO_4_, 0.033 mol/L NaH_2_PO_4_ (pH-value: 7.15 ± 0.05), 5% D_2_O, 6 mM pyrazine as an internal standard for quantification) were mixed. Then, 600 µL of the mixture was transferred to a 5 mm NMR tube (Bruker Corporation, Billerica, MA, USA). The samples were kept at 4 °C prior to measurement and analyzed within 24 h. NMR spectra of the serum were recorded at 310 K using an AVANCE NEO spectrometer (Bruker Corporation) operating at a proton frequency of 600 MHz. Spectra were recorded using a NOESY pulse sequence with water pre-saturation “noesygppr1d” in Bruker notation. For each sample, 16 subsequent scans were collected with a 12 s relaxation delay, an acquisition time of 2.75 s, 96 k data points, and a spectral width of 30 ppm. The spectra were processed, and 102 blood serum metabolites were quantified using Lifespin’s proprietary profiling software version 1.4 (Lifespin GmbH, Regensburg, Germany). The concentration of any NMR-measured metabolite was obtained as a signal integral of non-overlapping resonances or a cluster of partly overlapping resonances. The metabolite resonances were identified according to chemical shift assignments using Lifespin’s proprietary substance reference database (Lifespin GmbH, Regensburg, Germany).

The metabolomic data was analysed using R 4.1.3. Features with over 20% of values below limits of detection (LOD) were discarded. Remaining values below LOD were set to LOD/2. Approximate normality of metabolite concentrations was assessed by plotting their probability density functions. Two metabolites (Isopropanol, Unknown-Methane) were subsequently removed because of extreme distributional abnormality. The ‘limma’ package was used to investigate the effect of taking a liver biopsy and the effect of the viral load after inoculation on the metabolome of the ponies by use of linear models accounting for the ponies as random effects. *p* values were adjusted using the method of Benjamini and Hochberg [24].

#### 2.5.6. RNA Extraction and RNA Sequencing (RNA-seq) of Liver Tissue

Liver biopsies were flash-frozen in liquid nitrogen after collection and subsequently stored at −80 °C, prior to further processing at the VetCore Facility for Research, University of Veterinary Medicine Vienna. Liver biopsies were mechanically homogenized on a MagNA Lyser instrument (Roche, Rotkreuz, Switzerland) using 1.4 mm ceramic beads (Qiagen, Hilden, Germany) at 6500 rpm for 30 s. RNA extraction was done with the RNeasy Mini Kit (Qiagen) according to the recommended protocol for animal tissues with one modification: 50% instead of 70% ethanol was added to the homogenized lysate at step 4 of the protocol. RNA integrity was assessed with a 4200 TapeStation using the RNA ScreenTape assay (Agilent, Santa Clara, CA, USA). 

Sequencing libraries were prepared with the SENSE mRNA Library Prep Kit V2 (Lexogen, Vienna, Austria) using 600 ng total RNA input. Library quality control was done with D1000 ScreenTape assay on the 4200 TapeStation (Agilent). Libraries were sequenced on two lanes of a NextSeq 550 platform (Illumina, San Diego, CA, USA) implementing single-read 75-base pair (bp) reads. Sequencing was performed at the next-generation sequencing (NGS) unit of the Vienna Biocenter Core Facilities (VBCF, Vienna, Austria). RNA-seq reads are publicly available at NCBI Sequence Read Archive (SRA) under the BioProject number PRJNA722119. Quality filtering of the raw sequence reads was performed with Trimmomatic v0.32 [25]. Adapter sequences, low quality bases (Phred score < 25) and reads shorter than 35 nucleotides (nt) were removed. RNA-seq data analysis was done with CLC Genomics Workbench 20.0.3 software (Qiagen Bioinformatics, Aarhus, Denmark). Filtered reads were mapped against the horse genome (EquCab3.0) using the CLC Genomics RNA-seq tool with the default mapping parameters. Read counts were converted to transcripts per million (TPM) for normalization [26]. Principal component analysis (PCA) was calculated in CLC Genomics to visualize the overall gene expression profiles. For all the RNA-seq data analyses, data from the vaccine ponies were always compared pairwise for the three biopsy time points (day +13 vs. day −63; day +97 vs. day +13; day +97 vs. day −63). Based on evidence for an apparent failure of the vaccine in Vaccine Pony 4, the animal was excluded from the differential expression analysis. Similarly, data from the control ponies were always compared pairwise for the three biopsy time points. For all pairwise analysis of biopsy samples taken at different times, comparison of the later time point was made with the earlier time point. Differentially expressed genes (DEGs) within groups and between time points were identified by using absolute fold changes in expression > 2, false discovery rate-adjusted (FDR-adjusted) *p*-values < 0.05, and mean reads per kilobase of transcript per million reads mapped (RPKM) values > 0.5 [27]. DEGs were considered as interferon-stimulated genes (ISGs) when found upregulated (FDR < 0.05) in the “Orthologous Clusters of Interferon-Stimulated Genes” database [http://isg.data.cvr.ac.uk/; accessed on 12 May 2022] for the species horse [28]. The lists of DEGs were analysed for enriched Gene Ontology (GO) terms from the category “biological process” (BP) using the Cytoscape plug-in ClueGO v2.5.7 [29]. Bonferroni corrected *p* values < 0.05 were considered as significant. The option “GO term fusion” was used to group enriched terms based on the overlap of the associated genes. REVIGO [http://revigo.irb.hr/; accessed on 12 May 2022] was used to reduce the redundancy of the enriched BP GO terms [30] using the following settings: allowed similarity “medium”, selected species “Whole UniProt database” and semantic similarity measure “SimRel”. ShinyGO v0.76 was used to find enriched pathways from the Kyoto Encyclopedia of Genes and Genomes (KEGG) database and to perform hierarchical clustering [31,32]. The following settings were used: species “best matching species”, pathway DB “KEGG”, *p*-value cut-off (FDR) “0.05” and number of top pathways to show “30”.

#### 2.5.7. Histopathology, Immunohistochemistry (IHC), and Fluorescent in Situ Hybridization (FISH) of Liver Tissue

Liver biopsies were placed into 10% neutral-buffered formalin and subsequently embedded in paraffin. Paraffin-embedded biopsies were analysed at the Institute of Pathology, University of Veterinary Medicine Hanover, Germany, by means of histopathology, IHC, and FISH analyses. 

Histopathological analysis was performed on slides stained routinely with haematoxylin and eosin (H&E). Periportal inflammatory cell infiltrates were characterized by using IHC, as described previously [6]. Briefly, IHC was performed using the avidin–biotin complex (ABC) method and antibodies directed against myeloid/histiocyte antigen (1:500, mouse monoclonal antibody MAC387, DakoCytomation), CD3 (1:1000, rabbit polyclonal antibody, DakoCytomation), and Pax-5 (1:100, mouse monoclonal antibody 24/Pax-5, BD Transduction). All reactions included a heat-induced antigen retrieval (microwave, 800 W) with citrate buffer for 20 min. The analysis of each staining was performed by counting the total number of immunostained cells in periportal areas per total liver biopsy area. In addition, the relative proportion of each inflammatory cell type was calculated by dividing the number of cells immunopositive for specific cell markers by the total number of the immunopositive cells in the periportal areas.

FISH for detection of EqHV was performed using the ViewRNA^TM^ ISH Tissue Core Kit (Invitrogen by Thermo Fisher Scientific, Vienna, Austria) as described previously [7,33], with minor variations. EqHV-specific sequences were visualized with probes designed to specifically hybridize to the EqHV NS3-sequences (Affymetrix Inc. Santa Clara, CA, USA). For evaluation, five randomly selected pictures were taken of each sample and compared to the same localizations within the nonprobe control of the respective sample. All pictures were taken at the same magnification and with the same exposure time. Comparative analysis was performed with Fiji (ImageJ 1.52p) by generating the percentage of positive area. For elimination of unspecific background reactions, the percentage of positive area of negative controls was subtracted from the respective area of slides with probe application.

During analysis of the biopsies, investigators were blinded to the EqHV vaccination status of ponies (vaccine vs. control) and stage of EqHV infection (reference/day −63; early infection/day +13; or late infection/day +97) when biopsies were collected. 

#### 2.5.8. Detection of EqHV RNA in Liver Tissue 

RNA extracted from liver biopsies (as described above) was frozen at −80 °C, prior to further processing at the Department of Molecular and Medical Virology, Ruhr University Bochum, Germany. As determined using a NanoDrop One Microvolume UV Spectrophotometer (ThermoFisher), 50 ng of total RNA per reaction were subjected to one-step SYBR Green based qPCR using GoTaq^®^ qPCR Master Mix as described above. The LLOQ of this qPCR was 1 × 10^3^ viral copies/50 ng total RNA. All RNA samples from liver biopsies were analysed in duplicate, except for the biopsy from Vaccine Pony 3 on day +97, for which only a single analysis was done. The mean viral load of the two sample duplicates was determined for each biopsy. 

## 3. Results

### 3.1. EqHV RNA in Serum of Vaccine Ponies and Control Ponies

**Figure 1:**
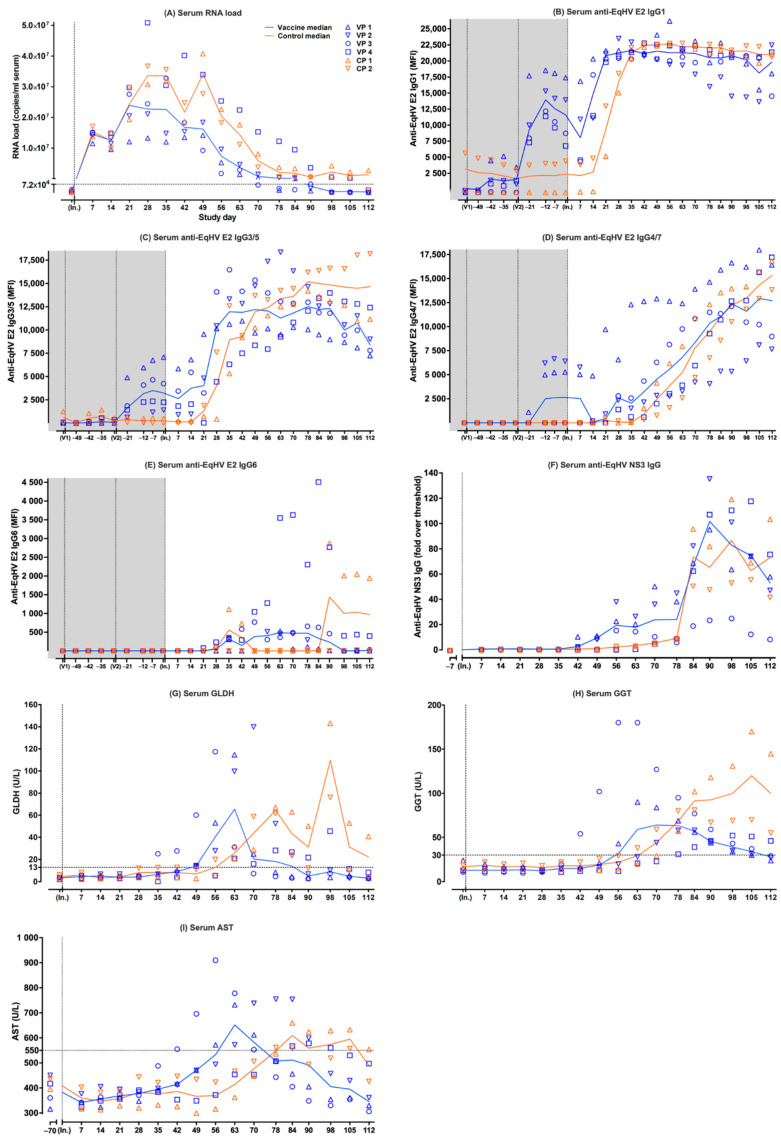
Individual (symbols) and median (lines) values of parameters measured in serum samples of six experimental ponies over time. Data from the vaccine ponies (VP) are shown in blue and data from the control ponies (CP) are shown in orange. Prime (V1) and booster (V2) vaccinations were administered on day −55 and day −27, while viral inoculation (In.) occurred on day 0. The parameters measured were (**A**) serum EqHV RNA load (LLOQ of the EqHV qPCR = 7.2 × 10^4^ viral copies/mL of serum); (**B**) serum anti-EqHV E2 IgG1; (**C**) serum anti-EqHV E2 IgG3/5; (**D**) serum anti-EqHV E2 IgG4/7; (**E**) serum anti-EqHV E2 IgG6; (**F**) serum anti-EqHV NS3 IgG; (**G**) serum GLDH (reference < 13 U/L); (**H**) serum GGT (reference < 30 U/L); (**I**) serum AST (reference < 550 U/L). AST = aspartate aminotransferase; CP = Control Pony; EqHV = equine hepacivirus; GLDH = glutamate dehydrogenase; GGT = gamma-glutamyl transferase; IgG = immunoglobulin G; In. = viral inoculation; LLOQ = lower limit of quantitation; MFI = median fluorescent intensity; NS3 = nonstructural protein 3; RNA = ribonucleic acid; qPCR = quantitative polymerase chain reaction; V1 = vaccination 1; V2 = vaccination 2; VP = Vaccine Pony. In this figure, all serum EqHV RNA load values ≤ LLOQ (≤7.2 × 10^4^ viral copies/mL of serum) were indicated as ≤LLOQ. Serum AST baseline values were determined on day −70.

Following viral inoculation, EqHV RNA was detected in the serum of all six ponies on day +7. Earlier, lower peak median serum RNA load was observed in the vaccine ponies compared to the control ponies (Figure 1A). The peak median serum RNA load in the vaccine ponies (median = 2.40 × 10^7^ copies/mL serum; [range = 1.20 × 10^7^–2.97 × 10^7^]) was detected on day +21, while the peak median serum RNA load in the control ponies (3.42 × 10^7^ copies/mL serum; [range = 2.76 × 10^7^–4.07 × 10^7^]) was detected on day +49 (Figure 1A). The median time from peak serum RNA load to decrease of serum RNA load below the LLOQ in the vaccine ponies was 51.5 days [range = 35–84]. The median time from viral inoculation to decrease of the serum RNA load below the LLOQ in the vaccine ponies was 90 days [range = 70–112]. The last of the vaccine ponies to have their serum RNA load decrease below the LLOQ was Vaccine Pony 4 by day +112. The serum RNA load of Control Pony 2 only decreased below the LLOQ by day +112, while the serum RNA load of Control Pony 1 remained above the LLOQ at the end of data collection (Figure 1A). 

### 3.2. Anti-EqHV E2-Specific IgG Isotypes in Serum of Vaccine Ponies and Control Ponies

Following a low E2 IgG1 response to the first vaccination (day −55) in 3/4 vaccine ponies, a higher E2 IgG1 response was observed in 4/4 vaccine ponies after the second vaccination (day −27) (Figure 1B). Prior to viral inoculation, the median E2 IgG1 peaked (13,953.95 MFI; [range = 11,634.2–18,679.2]) in the vaccine ponies 15 days after the second vaccination (day −12) and declined rapidly afterwards. After viral inoculation, the median E2 IgG1 continued to decline in the vaccine ponies until day +14, when the start of a rapid increase was observed. The median E2 IgG1 plateaued between day +21 (20,821.75 MFI; [range = 19,931–21,679.5]) and day +90 (20,816.75 MFI; [range = 14,697.5–21,364.5]), before gradually starting to decline (Figure 1B). Control Pony 2 appeared to have low E2 IgG1 present prior to viral inoculation. After viral inoculation, E2 IgG1 started to increase rapidly in the control ponies from day +21, one week later than in the vaccine ponies. Between day +35 and day +42, the control ponies reached an equal median level of E2 IgG1 compared to the vaccine ponies. It took approximately two weeks longer for the control ponies to reach a median level of E2 IgG1 equal to that of the vaccine ponies after viral inoculation (Figure 1B). The median E2 IgG1 of the control ponies remained at a plateau between day +35 (21,317.25 MFI; [range = 20,489–22,145.5]) and day +112 (20,962.75 MFI; [range = 20,745.5–21,180]) and the values were relatively closely matched to those of the vaccine ponies during this time. 

Overall, the E2 IgG3/5 responses were more variable among the ponies than was observed for the E2 IgG1 responses. An E2 IgG3/5 response to the second vaccination (day −27) was observed in 4/4 vaccine ponies prior to viral inoculation (Figure 1C). The peak median E2 IgG3/5 after the second vaccination (3494.2 MFI; [range = 1169.2–6742.7]) was later (day −7) than was observed for E2 IgG1 in the vaccine ponies. Following this peak, 20 days after the second vaccination, median E2 IgG3/5 declined slowly in the vaccine ponies. After viral inoculation, E2 IgG3/5 started increasing again in the vaccine ponies between day +14 and day +28, albeit at a slower rate than was observed for E2 IgG1. Steady increases and stable E2 IgG3/5 levels were observed in the vaccine ponies over the following weeks, with the median E2 IgG 3/5 plateauing between day +35 (11,968.5 MFI; [range = 6310–16,469.5]) and day +90 (12,310.75 MFI; [range = 8980–13,980]) (Figure 1C). The E2 IgG3/5 response of Vaccine Pony 4 resembled that of the control ponies more than that of the other vaccine ponies, with a slower, steadier increase in E2 IgG3/5 over time, peaking only at day +90 (13,980 MFI). Control Pony 1 appeared to have low, declining E2 IgG3/5 present prior to viral inoculation. After viral inoculation, E2 IgG3/5 started increasing in the control ponies from day +21. Between day +49 and day +56, the control ponies reached an equal median level of E2 IgG3/5, compared to the vaccine ponies (Figure 1C). It took approximately two weeks longer for the control ponies to reach a median level of E2 IgG3/5 equal to that of the vaccine ponies after viral inoculation (Figure 1C). The median E2 IgG3/5 of the control ponies continued to increase steadily, until it plateaued between day +78 (15186 MFI; [range = 14,188–16,184]) and day +112 (14,661.75 MFI; [range = 11,163–18,160.5]). 

The E2 IgG4/7 responses among the individual ponies, especially among the vaccine ponies, were also more variable than was observed for the E2 IgG1 responses. An E2 IgG4/7 response to the second vaccination (day −27) was observed in only 2/4 vaccine ponies prior to viral inoculation (Figure 1D). After viral inoculation, an overall slow, steady, increasing trend was observed in the median E2 IgG4/7, starting in the vaccine ponies between day +21 and day +28, and in the control ponies from approximately day +42. Following this initial lag in the increase of E2 IgG4/7 in the control ponies, the median E2 IgG4/7 of the control ponies remained relatively closely matched to that of the vaccine ponies for the remainder of the monitoring period. The overall rate of E2 IgG4/7 increase was slower than was observed for both E2 IgG1 and E2 IgG3/5. The E2 IgG4/7 appeared to plateau or peak in 3/4 vaccines ponies (Vaccine Ponies 1, 2, and 3) from day +90 onwards, while E2 IgG 4/7 in Vaccine Pony 4 and the control ponies continued to increase. 

E2 IgG1, IgG3/5, and IgG4/7 remained detectable in all six ponies at the end of data collection. 

In contrast to the above E2 IgG isotypes, no E2 IgG6 responses were observed after vaccination. After viral inoculation, the E2 IgG6 responses observed were of low magnitude, variable duration, and only detected in the four vaccine ponies and in Control Pony 1 (Figure 1E). 

### 3.3. Anti-EqHV NS3-Specific IgG in Serum of Vaccine Ponies and Control Ponies

Serum from all ponies remained negative for NS3 IgG on day −7, prior to viral inoculation. Following viral inoculation, the median time to detection of NS3 IgG in the vaccine ponies was 42 days, compared to 56 days in the control ponies (Figure 1F). Vaccine Pony 4 was the last of the six ponies to show a NS3 IgG response from day +63 onwards. NS3 IgG remained detectable in all six ponies at the end of data collection (day +112).

### 3.4. Liver-Associated Serum Biochemistry Parameters

Following viral inoculation, a median glutamate dehydrogenase (GLDH) concentration above reference range (reference <13 U/L) was detected earlier in the vaccine ponies (day +49 = 14.21 U/L; [range = 13.95–60.04]) compared to the control ponies (day +63 = 25.71 U/L; [range = 21.13–30.28]) (Figure 1G). A lower peak median GLDH concentration in the vaccine ponies (65.41 U/L; [range = 20.82–114.7]) was detected earlier (day +63), compared to the higher peak in the control ponies (109.63 U/L; [range = 76.18–143.08]) detected later (day +98). Changes in the median GLDH concentration of the control ponies appeared to be biphasic, with an additional, lower peak on day +78 (64.35 U/L; [range = 61.7–66.99]). The median GLDH concentration of the vaccine ponies returned to within reference range (5.15 U/L; [range = 2.78–21.66]; reference < 13 U/L) by day +90, while the median GLDH concentration of the control ponies remained above reference range (22.23 U/L; [range = 3.75–40.7]; reference < 13 U/L) at the end of data collection. In 5/6 ponies, the return of the GLDH concentration to within reference range happened shortly before or coincided with the decrease in the serum EqHV RNA load to below the LLOQ. Both an increased GLDH concentration and a serum EqHV RNA load above the LLOQ, were still detected in Control Pony 1 at the end of data collection.

Following viral inoculation, a median gamma-glutamyl transferase (GGT) concentration above reference range (reference < 30 U/L) was detected earlier in the vaccine ponies (day +56 = 31.5 U/L; [range = 12–180]) compared to the control ponies (day +70 = 44 U/L; [range = 29–59]) (Figure 1H). A lower peak median GGT concentration in the vaccine ponies (64 U/L; [range = 23–127]) was detected earlier (day +70), compared to the higher peak median GGT concentration in the control ponies (120 U/L; [range = 70–170]) detected later (day +105). The median GGT concentration of the vaccine ponies returned to within reference range (28 U/L; [range = 24–46]; reference < 30 U/L) by day +112, while the median GGT concentration of the control ponies remained well above reference range (100 U/L; [range = 55–145]; reference < 30 U/L) at the end of data collection. In 5/6 ponies, the GGT concentration remained above reference range even after the serum EqHV RNA load decreased below the LLOQ. Both an increased GGT concentration and a serum EqHV RNA load above the LLOQ were still detected in Control Pony 1 at the end of data collection.

Following viral inoculation, a median AST concentration above reference range (reference < 550 U/L) was detected earlier in the vaccine ponies (day +63 = 652 U/L; [range = 454–778]) compared to the control ponies (day +84 = 609.5 U/L; [range = 559–660]) (Figure 1I). These values also represented the peak median AST concentrations in both groups, with the peak median AST concentration of the control ponies being slightly lower than that of the vaccine ponies. The median AST concentration of the vaccine ponies returned to within reference range (508 U/L; [range = 443–755]; reference < 550 U/L) by day +78, while the median AST concentration of the control ponies returned to within reference range (490 U/L; [range = 426–554]; reference < 550 U/L) by day +112. In 5/6 ponies, the return of the AST concentration to within reference range happened shortly before, coincided with, or happened shortly after the decrease in the serum EqHV RNA load to below the LLOQ. Both an increased AST concentration and a serum EqHV RNA load above the LLOQ were still detected in Control Pony 1 at the end of data collection (day +112).

The normalized data of the changes in serum EqHV RNA, E2 IgG1, E2 IgG3/5, E2 IgG4/7, E2 IgG6, NS3 IgG, GLDH, GGT, and AST, as recorded in the six individual ponies over time, are illustrated in Supplementary Figure S1A–F. Temporal changes in certain parameters (serum EqHV RNA, GLDH, GGT, AST, E2 IgG3/5, E2 IgG4/7, and NS3 IgG) recorded for Vaccine Pony 4 (Supplementary Figure S1D) more closely resembled that of Control Ponies 1 and 2 (Supplementary Figure S1E,F), as opposed to Vaccine Ponies 1, 2, and 3 (Supplementary Figure S1A–C). In Vaccine Pony 4, as well as in Control Ponies 1 and 2, declining serum EqHV RNA levels corresponded temporally with stable, peak levels of E2 IgG1, and increasing levels of E2 IgG3/5, E2 IgG4/7, and NS3 IgG (Supplementary Figure S1D–F). In Vaccine Ponies 1, 2, and 3, the earlier decline in serum EqHV RNA levels corresponded temporally with peak or already declining E2 IgG1 and E2 IgG3/5 levels, as well as increasing levels of E2 IgG4/7 and NS3 IgG (Supplementary Figure S1A–C).

### 3.5. Clinical Scoring 

The overall clinical scores of the control ponies were higher than that of the vaccine ponies (Table 1). The overall clinical scores were primarily determined by abnormal blood parameters (Table 1). The only pony for which any abnormal clinical parameters were recorded, was Vaccine Pony 3 (Table 1). A multiple correspondence analysis of the scores revealed that Vaccine Pony 1 to 3 clustered together, but Vaccine Pony 4 did not cluster with the other vaccinated ponies nor the control ponies.

### 3.6. Serum Metabolomics

**Figure 2:**
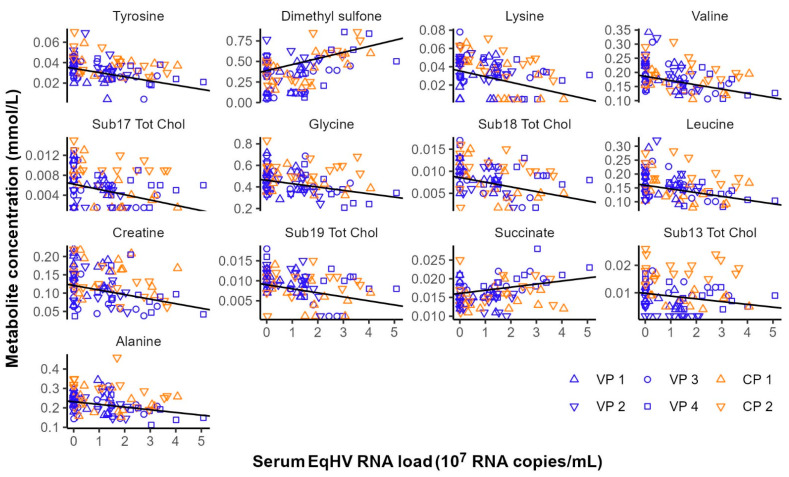
Metabolites significantly associated with equine hepacivirus (EqHV) RNA load (RNA copies/mL serum) in the serum samples of six ponies following experimental inoculation with EqHV polymerase chain reaction (PCR)-positive donor plasma. CP = Control Pony; RNA = ribonucleic acid; Sub.Tot.Chol = subgroup of total cholesterol; VP = Vaccine Pony.

Considering the effect of experimental interventions on serum metabolite concentrations, no metabolites were significantly associated with time (before vs. after vaccination) nor EqHV vaccination status (control ponies vs. vaccine ponies) when comparing samples collected before and after prime (day −56 vs. day −49) or booster (day −28 vs. day −21) vaccinations. The effect of EqHV inoculation was assessed by identifying serum metabolites significantly associated with serum EqHV RNA load. Eleven metabolites were shown to decrease in concentration in response to increasing serum RNA loads, while two metabolites were positively associated with the number of EqHV RNA copies per ml serum (Figure 2; Supplementary Table S2). Metabolites that were significantly associated with time (before vs. after liver biopsy), independent of EqHV vaccination status, when the data before and after the second liver biopsy (day +7 vs. day +14) and the third liver biopsy (day +90 vs. day +98) were analysed in combination, are listed in Table 2. Twenty-one metabolites decreased in concentration one day after liver biopsy, while three metabolites increased in concentration one day after liver biopsy (Table 2). No serum samples were collected one day after the first liver biopsy; therefore, this analysis included only data from serum samples collected one week before and one day after the second and the third liver biopsies.

### 3.7. RNA-seq of Liver Tissue

**Figure 3:**
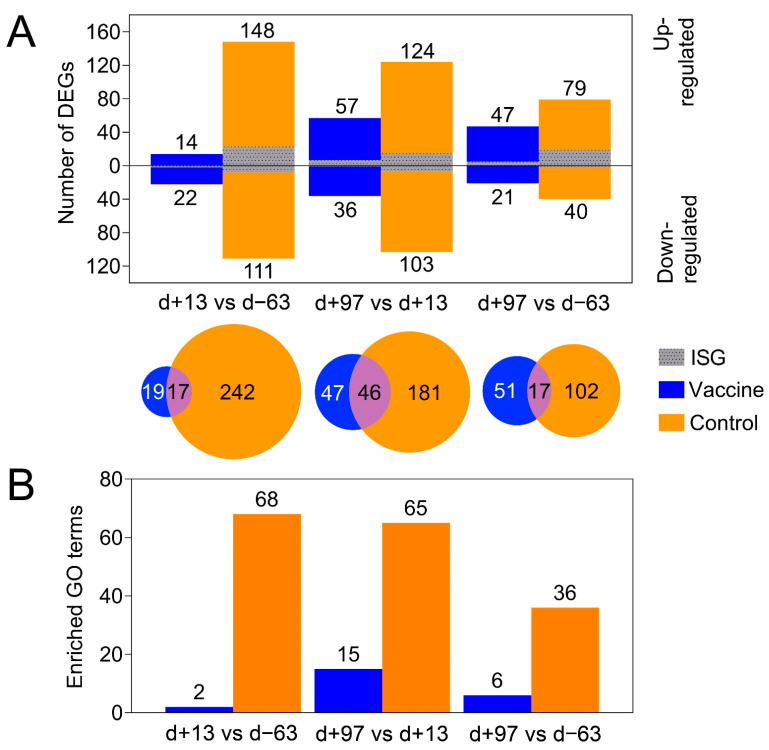
(**A**) The number of differentially expressed genes (DEGs) in the vaccine ponies (excluding Vaccine Pony 4, blue) and control ponies (orange) based on pairwise data comparisons for liver biopsy time points (day +13 vs. day -63; day +97 vs. day +13; day +97 vs. day -63). For all pairwise analysis of biopsy samples taken at different times, comparison of the later time point was made with the earlier time point. DEGs that were also identified as interferon stimulated genes (ISGs) are shown in grey. (**B**) The number of enriched gene ontology (GO) terms from the category “biological process” (BP) identified in the vaccine ponies (excluding Vaccine Pony 4, blue) and control ponies (orange) based on pairwise data comparisons for liver biopsy time points as described above. d = day; DEG = differentially expressed gene; GO = gene ontology; ISG = interferon stimulated gene.

**Figure 4:**
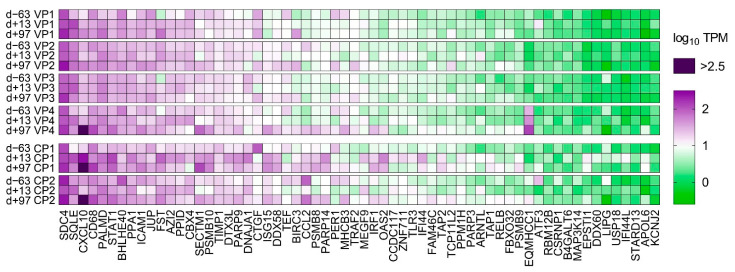
A heat map of normalized transcript expression (transcripts per million—TPM) of the 62 interferon stimulated genes (ISGs) identified in liver biopsies of the six individual ponies at the three different time points in relation to equine hepacivirus (EqHV) inoculation (day −63, day +13, day +97). CP = Control Pony; d = day; VP = Vaccine Pony.

**Figure 5:**
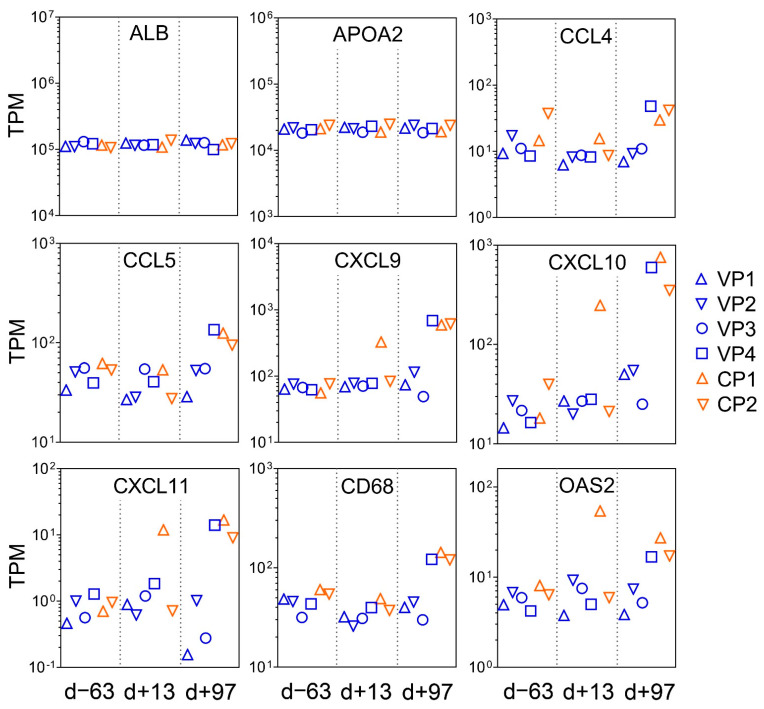
Normalized expression values (transcripts per million—TPM) for five inflammatory chemokines (CCL4, CCL5, CXCL9, CXCL10, CXCL11), one inflammation and macrophage marker gene (CD68), and an interferon-induced antiviral enzyme (OAS2), recorded in the liver biopsies of the six individual ponies at the three different time points in relation to equine hepacivirus (EqHV) inoculation (day -63, day +13, day +97). TPM values for albumin (ALB) and apolipoprotein A-II (APOA2) were also plotted as reference markers for liver tissue. CP = Control Pony; d = day; VP = Vaccine Pony.

PCA of the overall gene expression profiles showed a clear separation of both control ponies at day +13 and day +97 (Supplementary Figure S2). Notably, Vaccine Pony 4, which was excluded from differential expression analysis, clusters close to the control ponies at these respective time points. Few differences were found for the remaining ponies (Supplementary Figure S2). A greater number of DEGs were identified in the control ponies than in the vaccine ponies (excluding Vaccine Pony 4) for all three of the pairwise comparisons of time points (Figure 3A). Similarly, for all three of the pairwise comparisons of time points, a greater number of DEGs were identified as ISGs in the control ponies than in the vaccine ponies (Figure 3A). The complete list of DEGs for all pairwise comparisons is enclosed in Supplementary Table S3. Furthermore, analysis of the lists of DEGs for enriched GO terms also revealed a greater number of enriched GO terms in the control ponies than in the vaccine ponies for all three of the pairwise comparisons of time points (Figure 3B). The complete list of enriched GO terms for all pairwise comparisons is enclosed in Supplementary Table S4. With regards to expression of ISGs in the individual ponies at the different time points, a heat map of normalized transcript expression (TPM) of the 62 identified ISGs is shown in Figure 4. Increased expression of ISGs such as CXCL10, CD68, STAT1, and SECTM1 was detected in the liver biopsies of Control Ponies 1 and 2, as well as Vaccine Pony 4, during late EqHV infection (day +97) (Figure 4). Additionally, the TPM values for five inflammatory chemokines (CCL4, CCL5, CXCL9, CXCL10, CXCL11), one inflammation and macrophage marker gene (CD68), and an interferon-induced antiviral enzyme (OAS2), recorded in the liver biopsies from individual ponies at the different time points, were plotted in Figure 5. TPM values for albumin (ALB) and apolipoprotein A-II (APOA2) were also plotted as reference markers for liver tissue (Figure 5). Chemokine levels detected in the liver biopsy of Vaccine Pony 4 during late EqHV infection (day +97) closely resemble the increased chemokine levels detected in the late infection biopsies of Control Ponies 1 and 2, as opposed to the lower chemokine levels detected in the late infection biopsies of Vaccine Ponies 1, 2, and 3 (Figure 5). 

**Figure 6:**
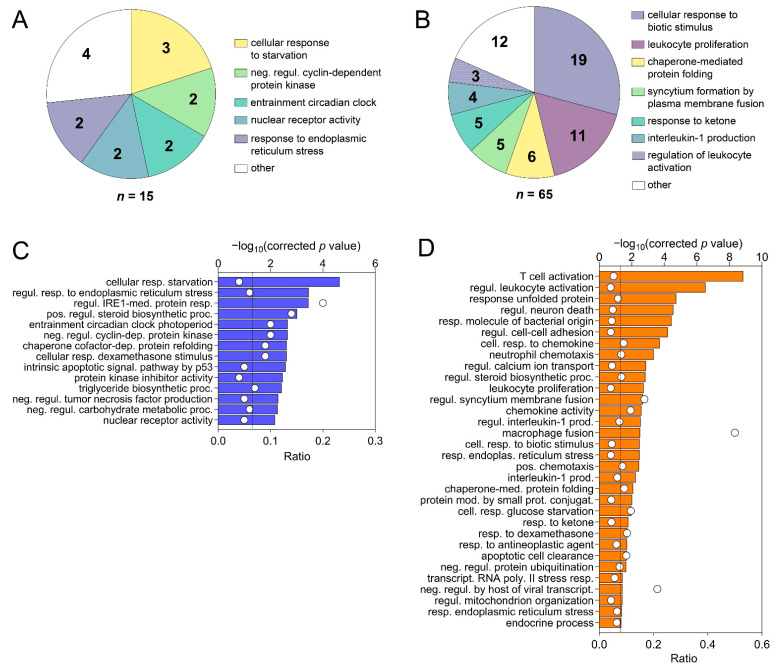
Grouping of all enriched Gene Ontology (GO) terms based on the overlap of associated genes as calculated by the “GO term fusion” option in ClueGO. Groups were named after the term with the lowest level in GO hierarchy. Numbers indicate the count of GO terms for each group for (**A**) the vaccine ponies (excluding Vaccine Pony 4, *n* = 15 terms) and (**B**) the control ponies (*n* = 65 terms), based on comparison of liver biopsies during late and early equine hepacivirus (EqHV) infection (day +97 vs. day +13). Nonredundant GO terms as filtered by “REVIGO” for (**C**) the vaccine ponies (excluding Vaccine Pony 4, *n* = 14 terms) and (**D**) the control ponies (*n* = 32 terms) for the comparison described above (day +97 vs. day +13), listed in descending order of corrected *p*-value. White circles (**C**,**D**) represent the ratio of differentially expressed genes (DEGs) from the data set compared to the total number of genes associated with the respective GO terms.

**Figure 7:**
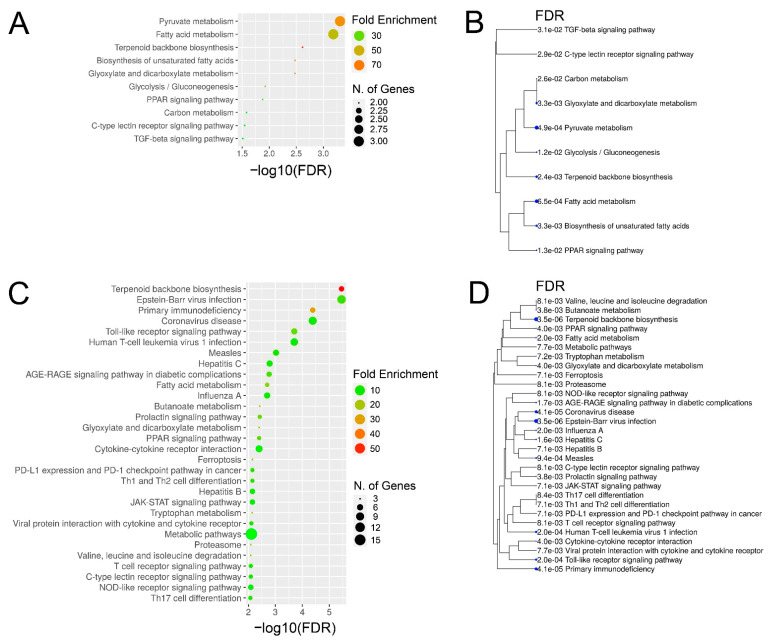
Enriched Kyoto Encyclopedia of Genes and Genomes (KEGG) pathways and hierarchical clustering tree for (**A**,**B**) the vaccine ponies (excluding Vaccine Pony 4, *n* = 10) and (**C**,**D**) the control ponies (*n* = 47), based on comparison of liver biopsies during late equine hepacivirus (EqHV) infection with biopsies collected prior to EqHV infection (day +97 vs. d -63). Enriched pathways (**A**,**C**) are listed in descending order of false discovery rate (FDR)-corrected *p*-value. Fold Enrichment refers to the percentage of genes in the sample list belonging to a pathway, divided by the corresponding percentage in the background. Only the 30 most significantly enriched pathways are shown for the control ponies (**C**,**D**).

Based on comparison of liver biopsies during late and early EqHV infection (day +97 vs. day +13), the GO terms for the vaccine ponies (excluding Vaccine Pony 4, *n* = 15 terms) and the control ponies (*n* = 65 terms) were grouped, and the most abundant GO groups were specified (Figure 6A,B). In contrast to the vaccine ponies, a larger number of GO groups were identified for the control ponies, and almost all of these groups were related to infection and immune responses (Figure 6B). Nonredundant GO terms for the vaccine ponies (excluding Vaccine Pony 4, *n* = 14 terms) and the control ponies (*n* = 32 terms), based on the comparison of liver biopsies on day +97 vs. day +13, were listed in descending order of corrected *p*-value (Figure 6C,D). In accordance with the observations of the GO term grouping, nonredundant GO terms in the control ponies were primarily related to cytokine, chemokine and immune cell responses, with “T-cell activation” and “regulation of leukocyte activation” being the terms with the highest corrected *p*-values (Figure 6D). Furthermore, 50% of genes associated with the term “macrophage fusion”, as well as 21.4% of genes associated with the term “negative regulation by host of viral transcription”, were differentially expressed in this comparison of liver biopsies from the control ponies (Figure 6D; Supplementary Table S4). In the vaccine ponies, terms such as “regulation of inositol-requiring enzyme 1 (IRE1)-mediated unfolded protein response” (20% of associated genes differentially expressed) could be related to viral infection, while terms such as “positive regulation of steroid biosynthetic process” (13.6% of associated genes differentially expressed) relate to metabolic processes (Figure 6C). Similar to observations related to enriched GO terms, comparison of liver biopsies during late EqHV infection with biopsies collected prior to EqHV infection (day +97 vs. d −63) also revealed more enriched KEGG pathways in the control ponies (*n* = 47) than in the vaccine ponies (*n* = 10) (Figure 7A–D). Enriched KEGG pathways were listed according to descending order of corrected *p*-value (Figure 7A,C) and hierarchical clustering (Figure 7B,D). Only the 30 most significantly enriched pathways are shown for the control ponies. Similar to observations related to enriched GO terms, enriched KEGG pathways in the control ponies were mostly related to immune responses and virus infections, including differential expression of more than 25% of genes associated with the HCV infection pathway (Figure 7C). In the vaccine ponies, the majority of enriched KEGG pathways were again related to metabolic processes (Figure 7A). 

### 3.8. Histopathology, IHC, FISH, and qPCR of Liver Tissue

**Figure 8:**
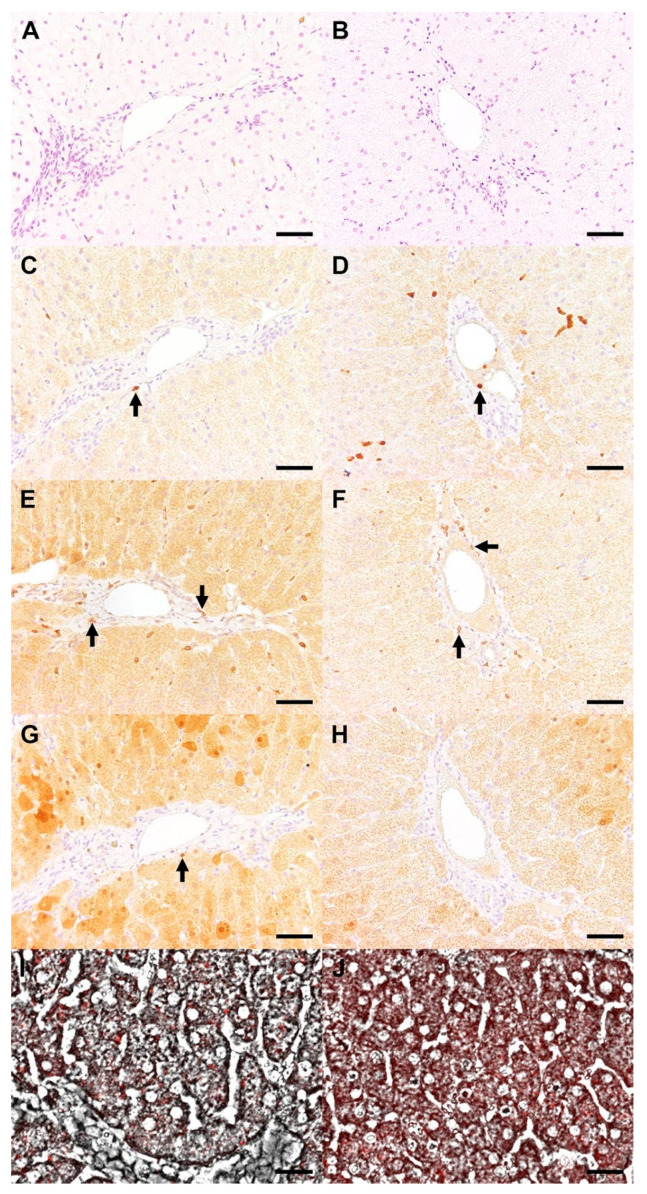
Liver biopsies from Control Pony 1 (**A**,**C**,**E**,**G**,**I**) and Vaccine Pony 1 (**B**,**D**,**F**,**H**,**J**) revealed a mild, periportal inflammatory cell infiltration in haematoxylin and eosin-stained samples (**A**,**B**). The infiltrate was composed of a few macrophages (arrows, (**C**,**D**)) and T lymphocytes (arrows, (**E**,**F**)). B lymphocytes were rarely observed (arrow, (**G**,**H**)). EqHV nucleic acid was distributed multifocally within the cytoplasm of hepatocytes, as demonstrated by fluorescent in situ hybridization/FISH (**I**,**J**). Bar = 50 µm (**A**–**H**), 25 µm (**I**,**J**).

Apart from minor individual variations, all 18 liver biopsies (*n* = 6 for day −63; *n* = 6 for day +13; *n* = 6 for day +97) had the following features in common: (1) mild, multifocal, periportally-accentuated, lympho-histiocytic inflammation (Figure 8A,B), and (2) mild, multifocal detection of a coarsely granular, yellow-brownish, cytoplasmic pigment in hepatocytes and Kupffer cells. Phenotyping of periportal inflammatory cells by means of IHC revealed the composition of the infiltrates to contain T lymphocytes > macrophages, with or without a small percentage of B lymphocytes (Figure 8C–H). The positive signal for detection of EqHV in liver biopsies by FISH was primarily located in the hepatocyte cytoplasm (Figure 8I,J). EqHV was detected in all liver biopsies (6/6) by qPCR during early infection (day +13) and in the biopsies of 2/4 vaccine ponies and 1/2 control ponies during late infection (day +97). EqHV qPCR results from liver biopsies post-infection (day +13 and day +97), as well as the corresponding serum EqHV qPCR results (day +14 and day +98) are reported in Table 3. 

## 4. Discussion

We investigated the immunogenicity and protective efficacy of an EqHV E2 recombinant protein candidate vaccine in ponies. A degree of variability was observed among the responses of individuals within both the vaccine and control groups. Although temporally separated, several recorded responses ultimately followed similar trends in both groups. However, the vaccine ponies appeared to have an earlier initial humoral immune response, to clear the EqHV infection earlier and to recover earlier from the EqHV-associated hepatic insult, compared to the control ponies. Differential expression of genes related to viral infection pathways, cytokine, chemokine, and immune cell responses was more pronounced in the control ponies compared to the vaccine ponies.

The vaccine ponies showed an earlier median peak serum EqHV RNA load, followed by an earlier decrease in median serum RNA load below LLOQ. The natural routes of horizontal EqHV transmission remain unknown, with respiratory transmission and arthropod-borne transmission being considered as possibilities [34,35,36]. Lower serum EqHV RNA load and earlier serum RNA clearance in vaccinated animals may also result in decreased risk of transmission. 

In equine serum, IgG4/7 is the most prevalent isotype, followed by IgG3/5, IgG1, and IgG6 [13]. Research into differential effector function capabilities of the IgG isotypes suggests that equine vaccine strategies should aim to elicit antibody responses of the IgG1, IgG3, IgG4, and IgG7 isotypes for maximum efficacy [13]. In the case of equine herpesvirus 1 (EHV-1), for which the IgG isotype responses to vaccination and challenge infection have been studied extensively, IgG4/7 and IgG1 levels correlated with serum neutralization titres [37]. In this study, E2 recombinant protein vaccination elicited E2 IgG1 and IgG3/5 responses in all the vaccine ponies. In all six ponies, the E2 IgG responses after viral inoculation consisted of an early, marked IgG1 response, followed relatively closely by a more variable IgG 3/5 response, and later by a delayed, variable IgG4/7 response. In all six ponies, the peak serum EqHV RNA load and the subsequent decrease in serum RNA corresponded with peak or near-peak IgG1, variable IgG3/5, and lower but increasing IgG4/7. This could be suggestive of IgG1 playing a role in initiating EqHV clearance. The role of IgG1 in EqHV infection clearance and the potential role of vaccine-induced IgG1 in accelerating EqHV infection clearance warrant further investigation. 

In an investigation of acute HCV infections in humans, immune responses were compared between patients who cleared the infection successfully (clearers) and patients who remained chronically infected (chronic progressors) [16]. Clearers had significantly earlier detectable E2 IgG1 responses and significantly earlier peak levels of E2 IgG1, although the magnitude of the E2 IgG1 responses did not differ significantly between the two groups [16]. Earlier, but not significantly different, onset and peak of the E2 IgG3 responses were also detected in clearers compared to chronic progressors [16]. During the decline of serum HCV RNA, potent E2 IgG1 and IgG3 responses were detected in all clearers, while these responses were low or absent in chronic progressors [16]. These findings provide insight into the potentially desirable IgG responses that future hepacivirus vaccine efforts ought to be aimed at.

Walker et al. further observed that, in clearers of HCV infection, E2 IgG responses occurred before or in close proximity to antibody responses to non-envelope proteins (i.e., anti-core, NS3, and NS5) [16]. In chronic progressors, E2 IgG responses were often delayed until after non-envelope antibody responses [16]. Monitoring of NS3 IgG after viral inoculation provided comparative insight into the immune responses of the ponies to a nonstructural viral protein. E2 IgG responses preceded the appearance of NS3 IgG in all six ponies. To date, investigation of the humoral immune response to EqHV infection has focused on NS3 IgG. Hence, seroconversion after EqHV infection has often been described as “delayed” [6,38]. Our results show that, in the case of E2 IgG, seroconversion does in fact occur relatively soon after EqHV inoculation, and the response appears to be accelerated by E2 recombinant protein vaccination. A report of EqHV serum RNA clearance prior to the appearance of NS3 IgG in one horse, while other horses remained EqHV RNA-positive despite the presence of NS3 IgG, supports the notion of other earlier immune responses resulting in EqHV RNA clearance [7]. Future investigation of the role of cell-mediated immune responses, in addition to humoral immune responses, in the clearance of acute EqHV infection and prevention of chronic disease progression, is warranted.

In addition to providing insights into equine host immune responses to E2 recombinant protein vaccination and subsequent EqHV inoculation, this study demonstrates a broad spectrum of parameters that may be assessed in an equine model for vaccination against a hepacivirus. With limited models for testing of vaccines and an incomplete understanding of protective immune responses as some of the major challenges hindering the development of an HCV vaccine [9], the findings of our study support further investigation of equids as a potentially useful model for future hepacivirus vaccination strategies. Natural and experimental EqHV infections are frequently subclinical and associated with transient elevation of concentrations of liver-associated enzymes [4,7,35,39]. Considering the subclinical nature of the infection, a multimodal approach, combining multiple monitoring techniques, is recommended to optimise the assessment of subclinical disease progression. In our study, a combination of serum metabolomics and liver-associated biochemistry parameters, as well as RNA-seq, histopathology, IHC, FISH, and qPCR of liver tissue was used to assess EqHV-associated liver injury.

A lower magnitude of increase and earlier return to reference range were observed for the median concentrations of liver-associated serum biochemistry parameters in vaccine ponies compared to control ponies. In five ponies, the return of serum GLDH concentrations to reference range preceded or coincided with the serum RNA decreasing below LLOQ. However, in all five of these ponies, serum GGT concentrations remained above reference range after serum RNA decreased below LLOQ. The delayed return of GGT to reference range could, at least partially, be attributed to the longer half-life of GGT compared to GLDH in horses. It could also be argued that this observation may have resulted from hepatocellular damage by the virus itself, followed by an inflammatory response that subsequently resulted in hepatobiliary damage. The limited number of liver biopsies collected after viral inoculation did not allow for confirmation of the latter possibility. Histopathology and immunohistochemistry findings of the liver biopsies did not differ remarkably from what is expected in horses of this age group, independent of underlying hepatic disease. Comparable histopathological findings have also been reported previously in horses that were experimentally infected with EqHV [6]. 

The decrease in concentrations of multiple amino acids, associated with increasing serum EqHV RNA load, may indicate involvement of the aminoacyl-tRNA synthetase (ARS) pathways [40]. ARSs do not only play a vital role in protein synthesis but are also known to play crucial roles in immune cell development, act as regulators and signaling molecules in various infectious diseases and have protective functions during viral infections [40]. The hepatocellular damage induced by liver biopsy appeared to disrupt homeostatic functioning and affect several pathways related to energy metabolism (pyruvate, lactate), lipoprotein metabolism (total cholesterol and subgroups, carbon chain elements), protein metabolism (histidine, glutamine), metabolism of ketone bodies (acetoacetate), and fluid balance (albumin). Comparison of the concentration of serum metabolites before and after liver biopsy to that of metabolites associated with serum EqHV RNA load, revealed that the effect of liver biopsy on metabolomic changes was approximately 10 times greater than that of the serum EqHV RNA load. The hepatocellular damage that was induced by liver biopsy and was reflected in the subsequent serum metabolomic changes was not mirrored in the serum GLDH concentrations. This is particularly clear when considering the absence of obvious changes in serum GLDH concentrations one day after the second liver biopsy (day +14). These findings demonstrate the potential limitations of serum GLDH as a marker for hepatocellular damage during a single event of short duration (e.g., liver biopsy), as opposed to ongoing hepatocellular damage (e.g., EqHV infection). It also demonstrates the sensitivity of serum metabolomic changes as markers for hepatocellular damage.

When comparing the liver biopsy RNA-seq results, a greater number of DEGs were identified as ISGs in the control ponies than in the vaccine ponies, for all three of the pairwise comparisons of time points. ISGs are known to play an integral role in suppression of HCV replication [41]. Similar to acute HCV infection in humans, an ISG signature was also identified in the liver of a horse following experimental EqHV infection [38]. Greater activation of hepatic processes related to infection and immune responses in the liver tissue of control ponies compared to the vaccine ponies, was reflected both in the greater number of relevant enriched GO terms and KEGG pathways, as well as the fold enrichment of these enriched terms and pathways. This was particularly apparent when comparing the results 97 days PI to the two earlier time points. The differential expression of genes related to infection and immune responses in the liver tissue of control ponies 97 days PI was contrasted by the differential expression of genes mostly related to metabolic processes in the liver tissue of vaccine ponies. The possibility cannot be excluded that comparable activation of hepatic infection and immune response processes in the vaccine ponies went undetected as a result of the extended period (84 days) between the collections of liver biopsies after viral inoculation. These findings could potentially signify earlier response to EqHV infection and earlier restoration of hepatic metabolic processes in the liver tissue of vaccine ponies. The need for better understanding of the role of systemic and local hepatic cell-mediated immune responses to EqHV infection and clearance was highlighted by “T cell activation” being the enriched GO term with the highest corrected *p*-value in the comparison of biopsies from late and early EqHV infection in control ponies. These data suggest that local lymphocyte populations might play crucial roles in viral clearance. Multiple enriched GO terms and KEGG pathways related to immune cell responses in the absence of distinct, related histopathological changes in hepatic tissue may warrant further investigation. 

Vaccine Pony 4 had the highest serum RNA load for several weeks after viral inoculation and was the last vaccine pony to have the serum RNA load decrease below LLOQ. Following viral inoculation of Vaccine Pony 4, temporal response patterns of the liver-associated biochemistry parameters and E2 IgG isotypes resembled those of Control Ponies 1 and 2 rather than those of other vaccine ponies. Furthermore, increased levels of CCL4, CCL5, CXCL9, CXCL10, CXCL11, CD68, and OAS2 in the liver biopsies of Vaccine Pony 4 and the control ponies on day +97 corresponded with peak or near-peak serum levels of GLDH, GGT, and AST. The reasons for the apparent failure of the vaccine in Vaccine Pony 4 are not known. Investigation of the EqHV viral intrahost population composition of the genomic regions encoding E1 and E2 in experimentally infected horses revealed low bottleneck severity and high heterogeneity of the virus populations initiating infection [42]. Furthermore, intra-host viral diversification was observed over time [42]. Although intra-host heterogeneity of EqHV was shown to be less pronounced than for HCV [42], the potential roles in reducing vaccine effectiveness and inducing varied immune responses among hosts warrant consideration.

Overall, the EqHV E2 recombinant protein candidate vaccine merits further investigation and may prove useful in the development and optimization of new vaccine strategies, eliciting more effective immune responses. Future strategies may include the evaluation of heterologous prime-boost vaccination, combining different vaccines during the prime and boost phases and targeting the same or different antigens. The use of NS3 and NS5 antigens could be a promising strategy to increase the activation of antigen-specific T cells. 

Although the small number of ponies in the vaccine and control groups limited statistical interpretation of results, this feasibility study served as a proof of concept. The number of animals used was considered the minimum number to meaningfully address the study aims and sufficient to demonstrate the proof of concept. The available sample material was optimally utilised, and results of this study lay the groundwork for future investigations of hepacivirus immune mechanisms and vaccination strategies. Data from this study can be used for future sample size calculations for similar study approaches. Despite no detectable NS3 IgG in any of the ponies at commencement of the study, E2 IgG isotypes appeared to be present in the two control ponies prior to viral inoculation. This finding raises the question about potential previous EqHV infection and whether prior infection may have played a role in the observed host responses. According to Tomlinson et al., the clearance of a primary EqHV infection resulted in broad, nonsterilizing immunity and partial immune protection that lasted at least one year [38]. The possibility that low MFI values recorded in the control ponies prior to viral inoculation may have resulted from high assay background or prior exposure to other hepaciviruses, however, cannot be excluded. The EqHV E2 IgG assay was specifically established for the analysis of these study samples and could be used to detect EqHV E2 IgG in experimental horses prior to enrollment in future EqHV studies. Finally, readers and authors should be mindful of advances in the continuously developing field of equine viral hepatitis, especially the identification of EqPV-H in 2018 [43]. In most studies related to EqHV that were published prior to 2018, horses were not tested for concurrent EqPV-H infection. When such concurrent infections are not definitively ruled out, influences of EqPV-H on observations of earlier EqHV studies cannot be excluded.

## 5. Conclusions

In conclusion, although vaccination with an EqHV E2 recombinant protein did not result in complete protective immunity against experimental EqHV inoculation, the majority of vaccinated ponies cleared the serum EqHV RNA earlier than the control ponies. The majority of vaccinated ponies appeared to recover from the EqHV-associated liver insult earlier than the control ponies. The equine model shows promise as a surrogate model for future hepacivirus vaccine research.

## Figures and Tables

**Table 1:** The overall clinical scores calculated for the vaccine ponies (*n* = 4) and control ponies (*n* = 2) based on blood parameters (albumin, AST, GLDH, GGT, bilirubin, bile acids, triglycerides, serum iron, serum amyloid A, haematocrit, and leukocytes) and clinical parameters (heart rate, respiratory rate, and rectal temperature) recorded during the study period. D = day.

Blood Parameters Score Overview						
	Vaccine Pony 1	Vaccine Pony 2	Vaccine Pony 3	Vaccine Pony 4	Control Pony 1	Control Pony 2
D+7	0	0	0	1	1	0
D+14	1	0	0	1	2	4
D+21	0	0	0	1	0	0
D+28	0	4	0	0	1	0
D+35	0	0	1	0	1	0
D+42	0	0	3	1	1	0
D+49	1	2	5	1	3	0
D+56	4	1	5	0	0	1
D+63	5	5	4	1	1	3
D+70	3	4	3	1	2	4
D+78	1	4	2	2	3	5
D+84	1	3	1	3	5	5
D+90	1	2	2	4	6	3
D+98	2	1	1	4	5	3
D+105	1	1	1	2	5	3
D+112	0	0	1	1	5	2
Total blood parameters score	20	27	29	23	41	33
+ Total clinical parameters score	0	0	2	0	0	0
**Total overall clinical score**	**20**	**27**	**31**	**23**	**41**	**33**

**Table 2:** Metabolites significantly associated with time (before vs. after liver biopsy), independent of EqHV vaccination status, when the data before and after the second liver biopsy (day +7 vs. day +14) and the third liver biopsy (day +90 vs. day +98) were analysed in combination. Negative mean differences indicate a decrease in metabolite concentration following the biopsy. Positive mean differences indicate an increase in metabolite concentration following the biopsy. Adj. *p* value = adjusted *p* value; Sub.Tot.Chol. = subgroup of total cholesterol; Tot.CCE = total carbon chain elements; Unsat.CCE = unsaturated carbon chain elements.

Metabolite	Mean Difference	*t*	*p* Value	Adj. *p* Value
Pyruvate	0.027	5.334	0.000	0.001
Acetoacetate	−0.007	−5.258	0.000	0.001
Formate	−0.013	−5.177	0.000	0.001
Methanol	−0.058	−5.153	0.000	0.001
Albumin	−0.168	−3.647	0.001	0.017
Tot.CCE	−12.367	−3.532	0.002	0.018
Total protein	−0.064	−3.458	0.002	0.018
Carbon chains	−2.594	−3.443	0.002	0.018
Sphingomyelins	−0.175	−3.283	0.003	0.023
Sub8.Tot.Chol.	−0.024	−3.087	0.005	0.031
Fatty acids	−0.514	−3.072	0.006	0.031
Sub7.Tot.Chol.	−0.023	−3.039	0.006	0.031
Triglycerids	−0.203	−2.946	0.008	0.032
Multi unsat.CCE	−0.652	−2.899	0.008	0.032
Histidine	−0.013	−2.871	0.009	0.032
Alpha unsat.CCE	−1.477	−2.842	0.010	0.032
Glutamine	−0.091	−2.829	0.010	0.032
Unsat.CCE	−2.496	−2.820	0.010	0.032
Sub9.Tot.Chol.	−0.018	−2.817	0.010	0.032
Tot.cholesterol	−0.127	−2.737	0.012	0.035
Creatinine	−0.037	−2.719	0.013	0.035
Mannose	0.014	2.711	0.013	0.035
Lactate	0.425	2.579	0.017	0.045
Sub6.Tot.Chol.	−0.015	−2.537	0.019	0.047

**Table 3:** Equine hepacivirus (EqHV) quantitative polymerase chain reaction (qPCR) results from liver biopsies of six experimental ponies after EqHV infection (*n* = 6 on day +13; *n* = 6 on day +97), as well as the corresponding serum EqHV qPCR results (*n* = 6 on day +14; *n* = 6 on day +98) of these ponies. All ribonucleic acid (RNA) samples from liver biopsies were analysed in duplicate, except for the biopsy from Vaccine Pony 3 on day +97, for which only a single analysis was done ^c^. The mean viral load of the two sample duplicates was determined for each liver biopsy. Mean liver viral load values that were below the lower limit of quantitation (LLOQ = 1 × 10^3^ viral copies/50 ng total RNA) of this qPCR assay are indicated as <LLOQ. Serum viral load values that were below the lower limit of quantitation (LLOQ = 7.2 × 10^4^ viral copies/mL of serum) of this qPCR assay are indicated as <LLOQ.

	Mean Liver Viral Load (Viral Copies/50 ng Total RNA; LLOQ = 1 × 10^3^ Copies/50 ng Total RNA)	Serum Viral Load (Viral Copies/mL Serum; LLOQ = 7.2 × 10^4^ Copies/mL Serum)
	Day +13	Day +14
Vaccine Pony 1	8.72 × 10^4^	9.51 × 10^6^
Vaccine Pony 2	3.55 × 10^4^	1.14 × 10^7^
Vaccine Pony 3	7.52 × 10^4^	1.36 × 10^7^
Vaccine Pony 4	1.30 × 10^5^	1.47 × 10^7^
Control Pony 1	8.77 × 10^4^	1.50 × 10^7^
Control Pony 2	7.66 × 10^4^	9.80 × 10^6^
	Day +97	Day +98
Vaccine Pony 1	1.27 × 10^3^	<LLOQ
Vaccine Pony 2	<LLOQ	<LLOQ
Vaccine Pony 3	<LLOQ ^c^	<LLOQ
Vaccine Pony 4	1.00 × 10^4^	4.66 × 10^5^
Control Pony 1	9.28 × 10^3^	4.13 × 10^6^
Control Pony 2	<LLOQ	3.01 × 10^5^

## Data Availability

RNA-seq reads are publicly available at NCBI Sequence Read Archive (SRA) under the BioProject number PRJNA722119. The data presented in this study are available as supplementary material.

## Supplementary Materials

The following supporting information can be downloaded at: https://www.mdpi.com/article/10.3390/v14071401/s1. Supplementary Figure S1: Normalized data of the changes in serum EqHV RNA, EqHV E2 IgG1, EqHV E2 IgG4/7, EqHV E2 IgG3/5, EqHV E2 IgG6, EqHV NS3 IgG, GLDH, GGT, and AST, as recorded in six individual experimental ponies over time. Prime (V1) and booster (V2) vaccinations were administered on day -55 and day -27, while viral inoculation (In.) occurred on day 0. Data from the vaccine ponies (*n* = 4; Vaccine Pony 1, 2, 3, and 4) are shown in panels (A–D), respectively. Data from the control ponies (*n* = 2; Control Pony 1 and 2) are shown in panels (E,F), respectively. AST = aspartate aminotransferase; EqHV = equine hepacivirus; GLDH = glutamate dehydrogenase; GGT = gamma-glutamyl transferase; IgG = immunoglobulin G; In. = viral inoculation; NS3 = nonstructural protein 3; RNA = ribonucleic acid; V1 = vaccination 1; V2 = vaccination 2; Supplementary Figure S2: Principal component analysis (PCA) to visualize the overall gene expression profiles detected in the six individual ponies’ liver biopsies at the three different time points in relation to equine hepacivirus (EqHV) inoculation (day −63, day +13, day +97). d = day; Supplementary Table S1: Blood parameters and clinical parameters included in the calculation of an overall clinical score for six experimental ponies (*n* = 4 vaccine ponies; *n* = 2 control ponies) that were vaccinated, and subsequently experimentally inoculated with EqHV polymerase chain reaction (PCR)-positive donor plasma. AST = aspartate aminotransferase; GLDH = glutamate dehydrogenase; GGT = gamma-glutamyl transferase; Supplementary Table S2: Metabolites significantly associated with equine hepacivirus (EqHV) RNA load (RNA copies/mL serum) in the serum samples of six ponies following experimental inoculation with EqHV polymerase chain reaction (PCR)-positive donor plasma. The coefficient indicates the change in metabolite concentration (in mmol/l) associated with each additional copy of viral RNA, resulting in very small values. Adj. *p* value = adjusted *p* value; Sub.Tot.Chol. = subgroup of total cholesterol; Supplementary Table S3: The complete list of differentially expressed genes (DEGs) for pairwise comparisons of RNA sequencing (RNA-seq) data from liver biopsies. For all the RNA-seq data analyses, data from the vaccine ponies (excluding Vaccine Pony 4) were always compared pairwise for the three biopsy time points (day +13 vs. day −63; day +97 vs. day +13; day +97 vs. day −63). Similarly, data from the control ponies were always compared pairwise for the three biopsy time points. For all pairwise analysis of biopsy samples taken at different times, comparison of the later time point was made with the earlier time point. DEGs within groups and between time points were identified by using absolute fold changes in expression > 2, false discovery rate-adjusted (FDR-adjusted) *p*-values < 0.05 and mean reads per kilobase of transcript per million reads mapped (RPKM) values > 0.5; Supplementary Table S4: The complete list of enriched Gene Ontology (GO) terms for all pairwise comparisons of RNA sequencing (RNA-seq) data from liver biopsies. For all the RNA-seq data analyses, data from the vaccine ponies (excluding Vaccine Pony 4) were always compared pairwise for the three biopsy time points (day +13 vs. day −63; day +97 vs. day +13; day +97 vs. day −63). Similarly, data from the control ponies were always compared pairwise for the three biopsy time points. For all pairwise analysis of biopsy samples taken at different times, comparison of the later time point was made with the earlier time point. The lists of differentially expressed genes (DEGs) were analysed for enriched GO terms from the category “biological process” (BP) using the Cytoscape plug-in in ClueGO v2.5.7 [29]. Bonferroni corrected *p* values < 0.05 were considered as significant.
