# Supplementary material for: An Equine Model for Vaccination against a Hepacivirus: Insights into Host Responses to E2 Recombinant Protein Vaccination and Subsequent Equine Hepacivirus Inoculation
Source: Viruses. 2022 Jun 27;14(7):1401. doi: 10.3390/v14071401 (PMC9318657; doi:10.3390/v14071401)
Supplement: Supplementary file 1 [file viruses-14-01401-s001.zip › Supplementary Table S1.pdf]

Supplementary Table S1: Blood parameters and clinical parameters included in the calculation of an overall clinical score for six experimental ponies (n = 4 vaccine ponies; n = 2 control ponies) that were vaccinated, and subsequently experimentally inoculated with EqHV polymerase chain reaction (PCR)-positive donor plasma. AST = aspartate aminotransferase; GLDH = glutamate dehydrogenase; GGT = gamma-glutamyl transferase.

Parameter	Reference range	Score awarded	
**Blood parameters**			
Albumin	2.4 - 4.5 g/dl	<2.4 g/dl = 2	≥2.4 g/dl = 0
AST	<550 U/L	≥550 U/L = 1	<550 U/L = 0
GLDH	<13 U/L	>3 x 13 U/L (>39 U/L) = 2	1-3 x 13 U/L (13-39 U/L) = 1
GGT	<30 U/L	>3 x 30 U/L (>90 U/L) = 2	1-3 x 30 U/L (30-90 U/L) = 1
Bilirubin	0.7 - 3.1 mg/dl	>3.1 mg/dl = 1	≤3.1 mg/dl = 0
Bile acids	<20 umol/L	≥20 umol/L = 2	<20 umol/L = 0
Triglycerides	<50 mg/dl	>100 mg/dl = 2	50-100 mg/dl = 1
Serum iron	80 - 240 ug/dl	<80 ug/dl = 1	>240 ug/dl = 1
Serum amyloid A	<10 mg/L	>4 x 10 mg/L (>40 mg/L) = 2	2-4 x 10 mg/L (20-40 mg/L) = 1
Haematocrit	0.32 - 0.55	≥0.45 = 1	<0.3 = 1
Leukocytes	5000-10000/ul	>10000/ul = 1	<5000/ul = 1
**Clinical parameters**			
Heart rate	>48 beats/min	>48 beats/min = 1	
Respiratory rate	>28 breaths/min	>28 breaths/min = 1	
Rectal temperature	>38.5°C = 1	>38.5°C = 1	
