# Supplementary material for: An Equine Model for Vaccination against a Hepacivirus: Insights into Host Responses to E2 Recombinant Protein Vaccination and Subsequent Equine Hepacivirus Inoculation
Source: Viruses. 2022 Jun 27;14(7):1401. doi: 10.3390/v14071401 (PMC9318657; doi:10.3390/v14071401)
Supplement: Supplementary file 1 [file viruses-14-01401-s001.zip › Supplementary Table S2.pdf]

Supplementary Table S2: Metabolites significantly associated with equine hepacivirus (EqHV) RNA load (RNA copies/ml serum) in the serum samples of six ponies following experimental inoculation with EqHV polymerase chain reaction (PCR)-positive donor plasma. The coefficient indicates the change in metabolite concentration (in mmol/l) associated with each additional copy of viral RNA, resulting in very small values. (Adj. p value = adjusted p value; Sub.Tot.Chol. = subgroup of total cholesterol)

**Metabolite**	**Coefficient**	**t**	***p* value**	**adj. *p* value**
Tyrosine	-4.10×10^{-10}	-4.739	0.000	0.000
Dimethyl sulfone	7.40×10^{-09}	4.657	0.000	0.000
Lysine	-6.21×10^{-10}	-4.415	0.000	0.001
Valine	-1.51×10^{-09}	-3.896	0.000	0.003
Sub17.Tot.Chol.	-1.06×10^{-10}	-3.827	0.000	0.003
Glycine	-3.11×10^{-09}	-3.536	0.001	0.006
Sub18.Tot.Chol.	-1.06×10^{-10}	-3.478	0.001	0.007
Leucine	-1.34×10^{-09}	-3.383	0.001	0.008
Creatine	-1.24×10^{-09}	-3.352	0.001	0.008
Sub19.Tot.Chol.	-1.01×10^{-10}	-3.302	0.001	0.008
Succinate	8.27×10^{-11}	3.130	0.002	0.013
Sub13.Tot.Chol.	-1.00×10^{-10}	-3.068	0.003	0.014
Alanine	-1.38×10^{-09}	-3.006	0.003	0.016
