# Supplementary figures and images for: An Equine Model for Vaccination against a Hepacivirus: Insights into Host Responses to E2 Recombinant Protein Vaccination and Subsequent Equine Hepacivirus Inoculation
Source: Viruses. 2022 Jun 27;14(7):1401. doi: 10.3390/v14071401 (PMC9318657; doi:10.3390/v14071401)

### (A) Vaccine Pony 1

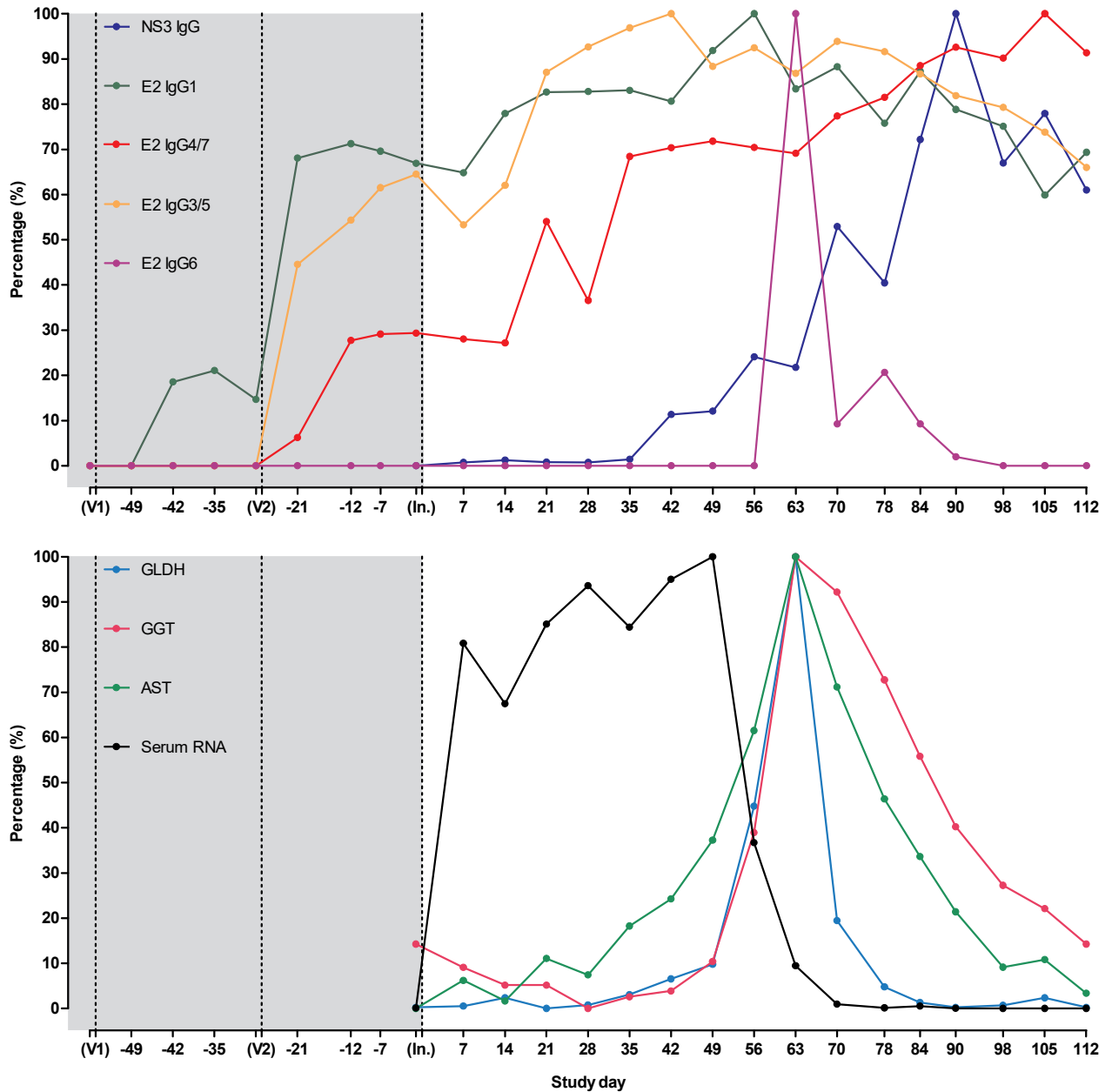

## (B) Vaccine Pony 2

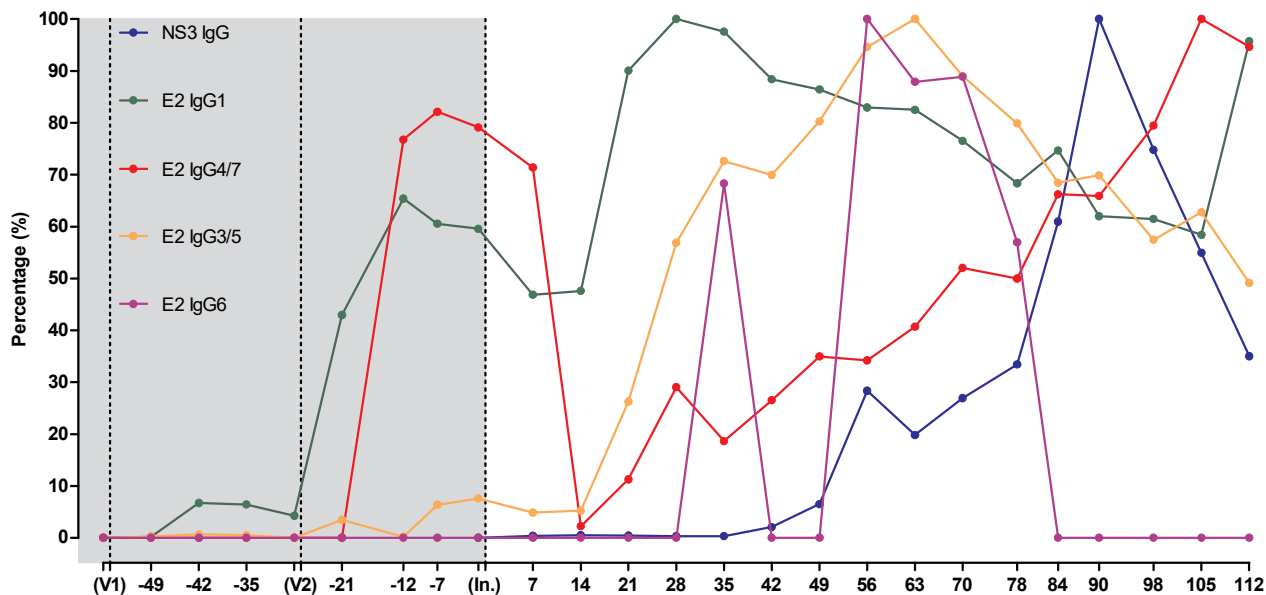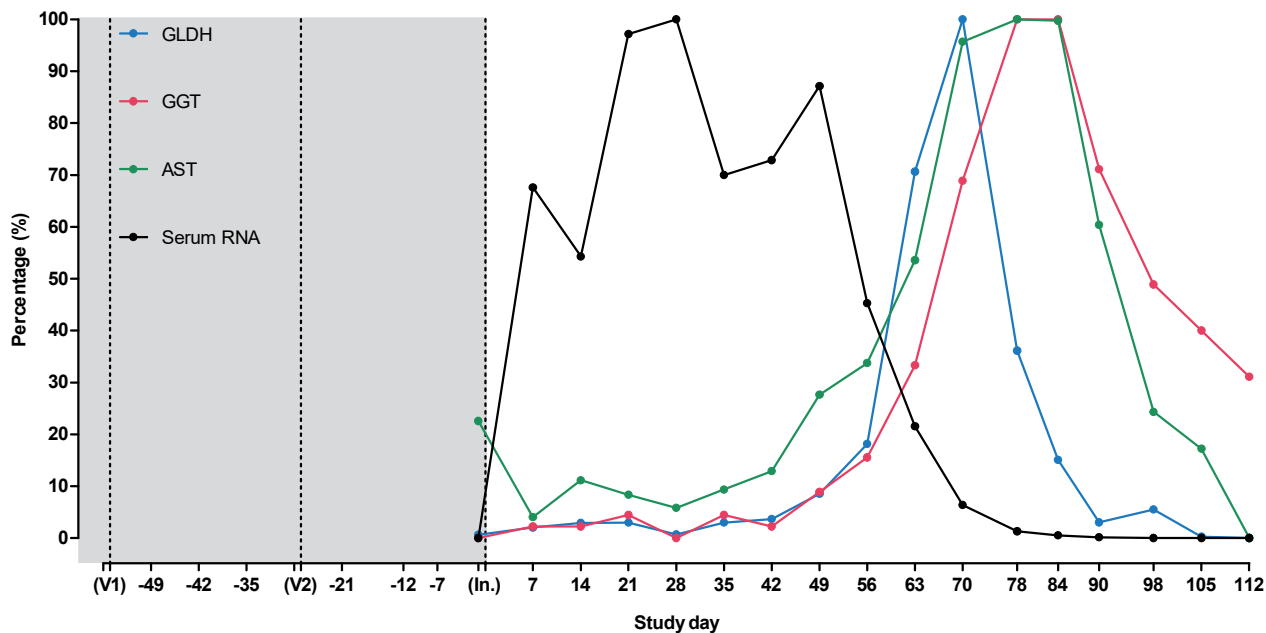

### (C) Vaccine Pony 3

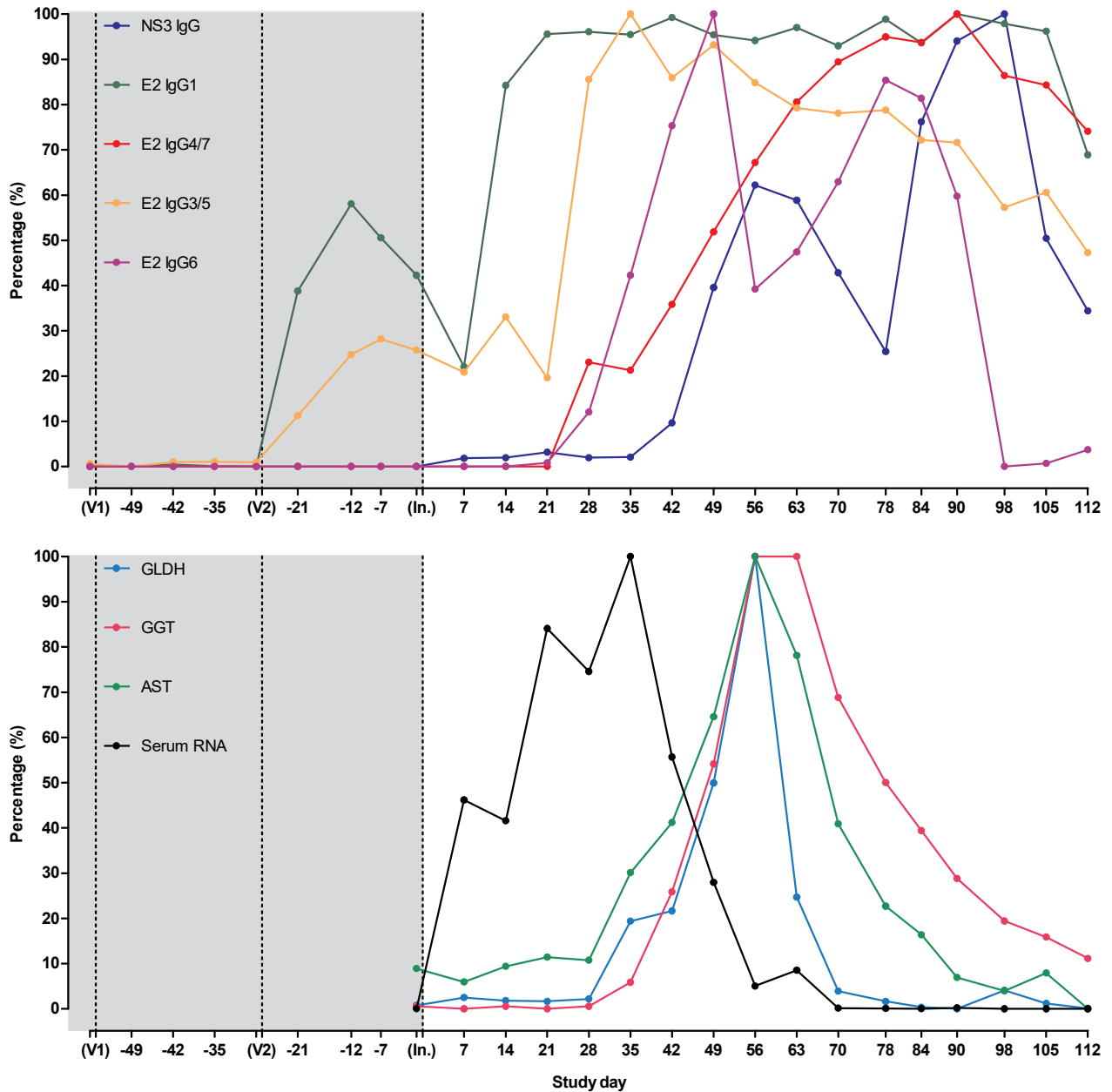

### (D) Vaccine Pony 4

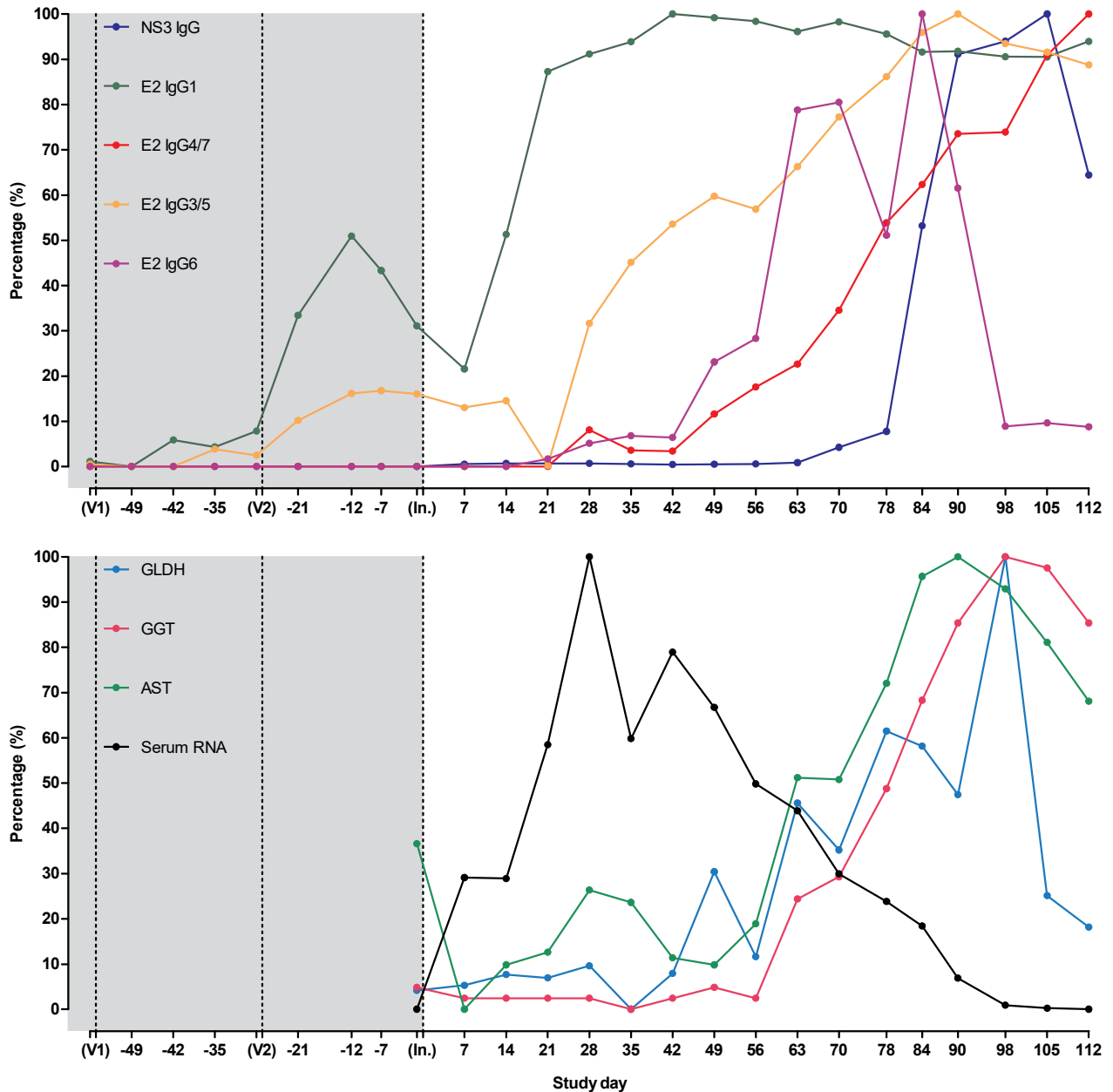

### (E) Control Pony 1

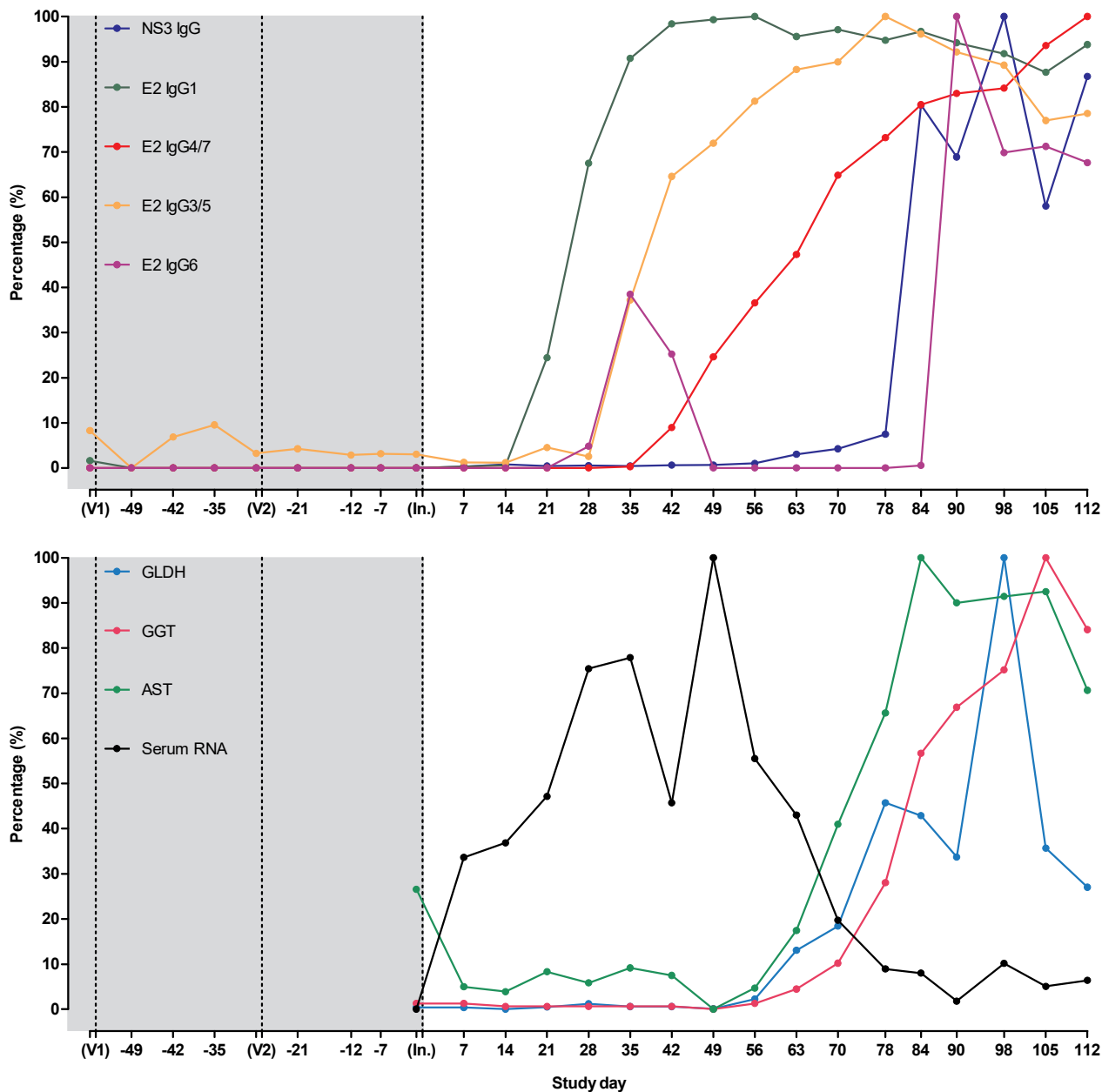

## (F) Control Pony 2

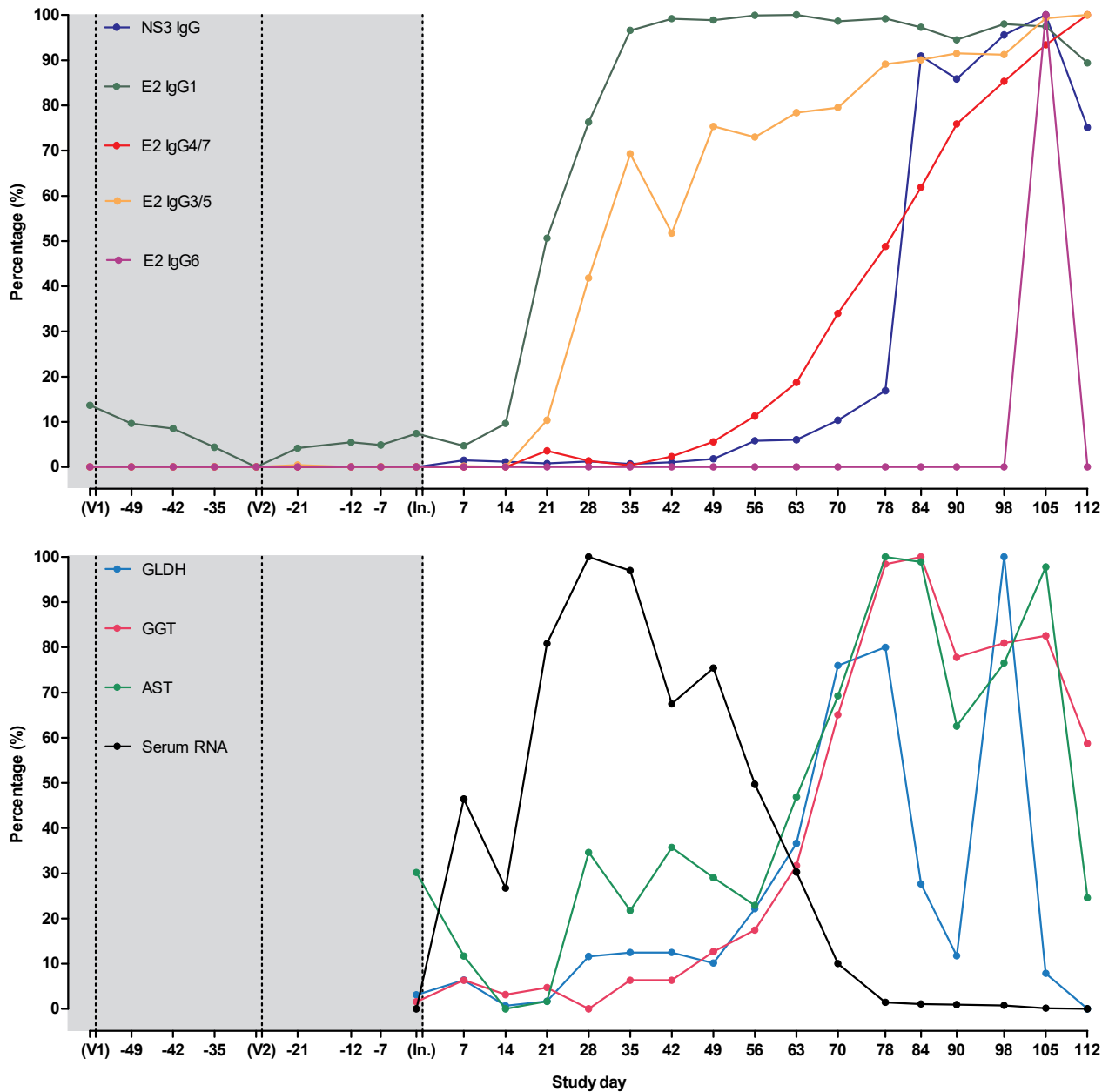

Supplement: Supplementary file 1 [file viruses-14-01401-s001.zip › Supplementary Figure S1.pdf]

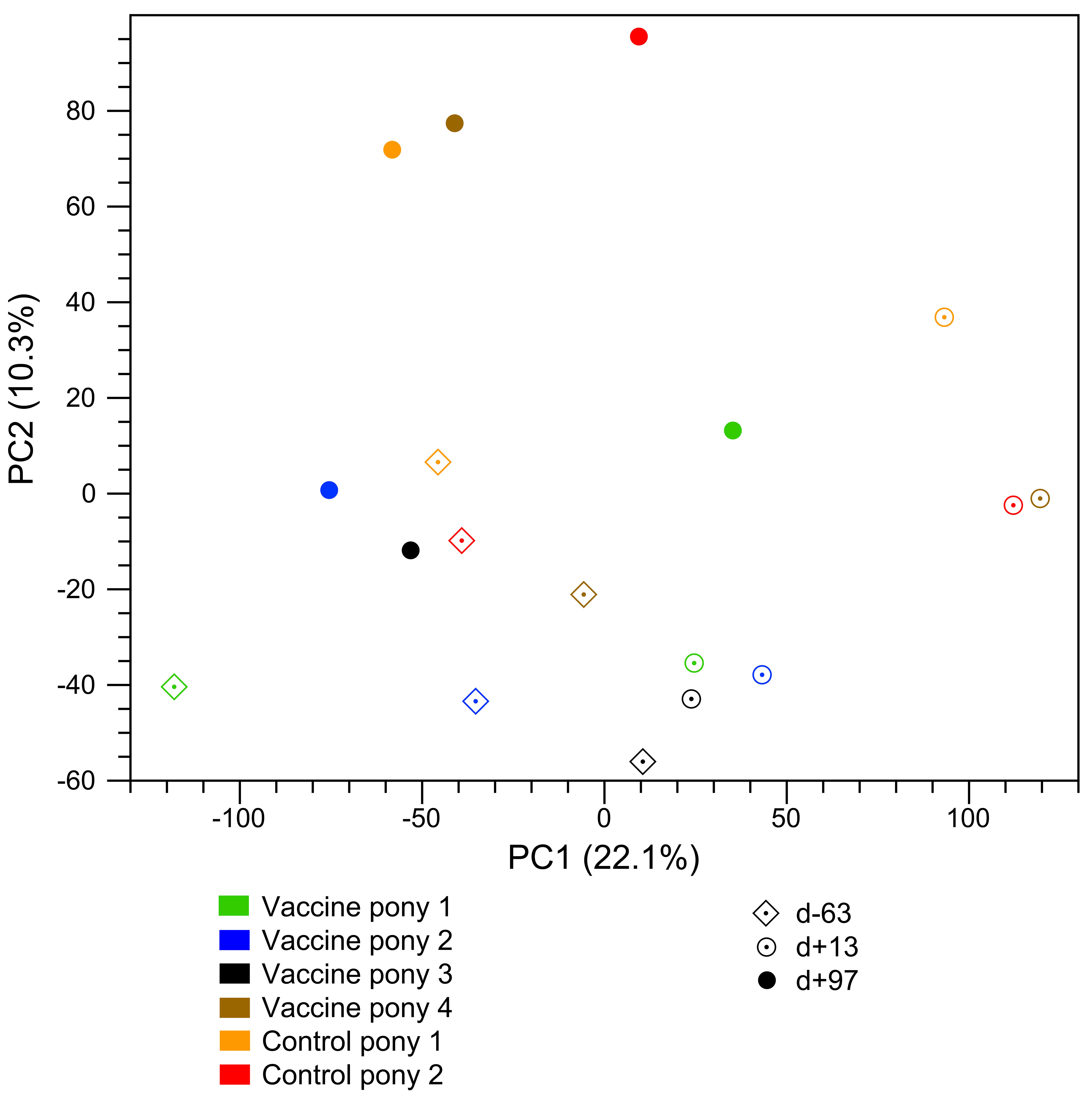

Supplement: Supplementary file 1 [file viruses-14-01401-s001.zip › Supplementary Figure S2 including VP4.jpg]
